# Messenger RNA transport on lysosomal vesicles maintains axonal mitochondrial homeostasis and prevents axonal degeneration

**DOI:** 10.1038/s41593-024-01619-1

**Published:** 2024-04-10

**Authors:** Raffaella De Pace, Saikat Ghosh, Veronica H. Ryan, Mira Sohn, Michal Jarnik, Paniz Rezvan Sangsari, Nicole Y. Morgan, Ryan K. Dale, Michael E. Ward, Juan S. Bonifacino

**Affiliations:** 1 Neurosciences and Cellular and Structural Biology Division, Eunice Kennedy Shriver National Institute of Child Health and Human Development, National Institutes of Health, Bethesda, MD USA; 2 Neurogenetics Branch, National Institute of Neurological Disorders and Stroke, National Institutes of Health, Bethesda, MD USA; 3 Bioinformatics and Scientific Programming Core, Eunice Kennedy Shriver National Institute of Child Health and Human Development, National Institutes of Health, Bethesda, MD USA; 4 Biomedical Engineering and Physical Science Shared Resource, National Institute of Biomedical Imaging and Bioengineering, National Institutes of Health, Bethesda, MD USA

**Keywords:** Cellular neuroscience, RNA, Lysosomes, Mitochondria

## Abstract

In neurons, RNA granules are transported along the axon for local translation away from the soma. Recent studies indicate that some of this transport involves hitchhiking of RNA granules on lysosome-related vesicles. In the present study, we leveraged the ability to prevent transport of these vesicles into the axon by knockout of the lysosome–kinesin adaptor BLOC-one-related complex (BORC) to identify a subset of axonal mRNAs that depend on lysosome-related vesicles for transport. We found that BORC knockout causes depletion of a large group of axonal mRNAs mainly encoding ribosomal and mitochondrial/oxidative phosphorylation proteins. This depletion results in mitochondrial defects and eventually leads to axonal degeneration in human induced pluripotent stem cell (iPSC)-derived and mouse neurons. Pathway analyses of the depleted mRNAs revealed a mechanistic connection of BORC deficiency with common neurodegenerative disorders. These results demonstrate that mRNA transport on lysosome-related vesicles is critical for the maintenance of axonal homeostasis and that its failure causes axonal degeneration.

## Abstract (web summary)

Using human iPSC-derived and mouse neurons, this study demonstrates that mRNA transport on lysosome-related vesicles is critical for the maintenance of axonal homeostasis and that its failure causes axonal degeneration.

## Main

Lysosomes are membrane-bound, acidic organelles that are primarily involved in macromolecular degradation but also play roles in other processes, including nutrient signaling and gene regulation^1,2^. Lysosomes are highly heterogeneous in size, shape, pH, acid hydrolase content and overall composition, reflecting different maturational states or specialized functions. Additionally, they share luminal and/or membrane components with late endosomes and various lysosome-related organelles. To encompass this diversity, herein we collectively refer to all these organelles as ‘lysosome-related vesicles’. Axonal lysosome-related vesicles are a prime example of such heterogeneity^3,4^. In the axon, lysosome-related vesicles move bidirectionally along microtubule tracks^5–13^. Movement toward the distal axon (that is, anterograde transport) is dependent on coupling to kinesin motor proteins, whereas movement toward the soma (that is, retrograde transport) is dependent on coupling to the dynein–dynactin motor complex^3,4^. In general, anterograde lysosome-related vesicles are smaller and less acidic than retrograde lysosome-related vesicles^5,12–14^. Moreover, at least some anterograde lysosome-related vesicles contain *trans*-Golgi network (TGN) and endosomal and synaptic vesicle proteins, suggesting that they serve as biosynthetic carriers or lysosome precursors^9,13^. The population of retrograde lysosome-related vesicles includes mature lysosomes, late endosomes, autolysosomes (fusion of lysosomes with autophagosomes) and/or amphisomes (fusion of lysosomes with late endosomes)^15–18^. The distinct properties of anterograde and retrograde lysosome-related vesicles suggest that these organelles undergo maturation in the distal axon.

Although anterograde lysosome-related vesicles most likely serve as precursors for mature lysosomes, they could perform additional functions along the way. One such function has recently come to light, with the discovery that RNA granules hitchhike on lysosome-related vesicles for long-distance transport along the axon^19^. RNA granules are phase-separated condensates of RNAs and RNA-binding proteins (RBPs) that enable regulation of RNA functions in various post-transcriptional processes^20^. RNA granules coupled to lysosome-related vesicles in the axon carry both miRNAs^21,22^ and mRNAs^19^ for regulated local translation at sites distal from the soma. Moreover, axonal late endosomes serve as platforms for local translation of nuclear-encoded mitochondrial mRNAs by associated ribosomes^23^. RBPs and mRNAs can also undergo axonal transport in association with mitochondria^24,25^ and early endosomes^23,26^ or through direct binding of RBPs to microtubule motors^27–29^. It is currently unknown whether each of these modalities is responsible for the transport of different subsets of RNAs.

**Fig. 1:**
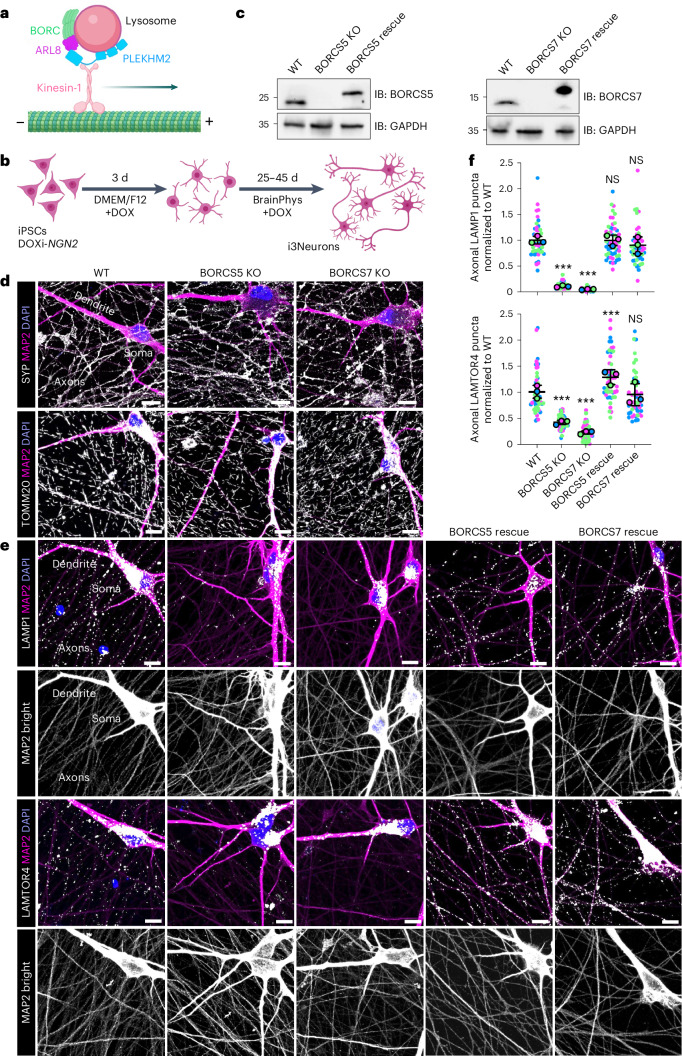
Depletion of axonal lysosomes in BORCS5-KO and BORCS7-KO i3Neurons. **a**, Schematic representation of the coupling of lysosomes to the plus-end-directed kinesin-1 motor through the BORC–ARL8–PLEKHM2 ensemble^31^. **b**, iPSCs were differentiated for 3 d in DMEM/F12 medium supplemented with NEAAs, GlutaMAX and N2A and doxycycline (DOX) and further cultured for 25–45 d in BrainPhys medium containing B27, laminin, BDNF, NT-3 and DOX. **c**, SDS-PAGE and immunoblot (IB) analysis of WT, BORCS5-KO, BORCS7-KO, BORCS5-KO re-expressing BORCS5-P2A-GFP (rescue) and BORCS7-KO re-expressing BORCS7-HA-P2A-GFP (rescue) iPSCs using antibodies to BORCS5, BORCS7 and GAPDH (loading control). After self-cleavage, 22 amino acid residues at the N-terminus of P2A remain linked to the upstream protein, thus the higher molecular mass in the rescue cells. The positions of molecular mass markers (in kDa) are indicated on the left. **d**,**e**, i3Neurons derived from the WT, BORCS5-KO, BORCS7-KO (**d**) and BORCS5 rescue and BORCS7 rescue (**e**) iPSCs shown in **c** were cultured for 25 d on glass coverslips and immunostained for endogenous MAP2 (soma and dendrites) (magenta), synaptophysin (SYP) (synaptic vesicles), TOMM20 (mitochondria), LAMP1 (lysosomes) or LAMTOR4 (lysosomes) (all grayscale). Nuclei were stained with DAPI (blue). Scale bars, 10 μm. See also Extended Data Figs. 1, 2a and 8a. Panel **e** additionally shows higher intensity images of MAP2 staining in grayscale (MAP2 bright). **f**, Quantification of the number of axonal puncta staining for LAMP1 or LAMTOR4 in various i3Neuron lines relative to WT i3Neurons (defined as 1.0) from *n* = 3 independent experiments such as that shown in **e**. Results are represented as SuperPlots^77^ showing the individual data points in different colors, the mean from each experiment and the mean of the means ± s.d. Statistical significance was calculated by one-way ANOVA with Dunnett’s multiple comparisons test. Axonal LAMP1 significance versus WT: BORCS5 KO ***P < 0.001, BORCS7 KO ***P < 0.001, BORCS5 rescue *P* = 0.995 and BORCS7 rescue *P* = 0.157. Axonal LAMTOR4 significance versus WT: BORCS5 KO ***P < 0.001, BORCS7 KO ***P < 0.001, BORCS5 rescue ***P < 0.001 and BORCS7 rescue *P* = 0.870. NS, not significant, relative to WT. Source data

Interrogating the role of axonal lysosome-related vesicles in RNA transport requires a method to halt movement of lysosome-related vesicles without altering other transport mechanisms. This discrimination would not be possible by silencing the motor proteins themselves, as this would have widespread effects on axonal transport. Instead, a more precise approach would be to silence a specific adaptor of lysosomes to kinesins. An adaptor system meeting this requirement is an ensemble of the BLOC-one-related complex (BORC), the small GTPase ARL8 and the adaptor protein PLEKHM2 (also known as SKIP), which mediates coupling of lysosomes to kinesins 1 and 3 (refs. ^30–32^) (Fig. 1a). BORC is a hetero-octameric protein complex composed of subunits named BORCS1–8 (ref. ^31^). Knockout (KO) of individual BORC subunits impairs anterograde transport of lysosome-related vesicles and causes their depletion from the peripheral cytoplasm in non-neuronal cells and from the axon in neurons^6,7,11,31^. These effects are specific, as the cytoplasmic or axonal distributions of other organelles, such as mitochondria and synaptic vesicles, are not affected^11,31^. Moreover, BORC-subunit KOs cause neonatal death in mice^7,11^, and hypomorphic mutations are associated with severe neurodevelopmental/neurodegenerative phenotypes in both mice^7^ and humans^33–35^. These findings indicate that BORC-dependent transport of lysosome-related vesicles is particularly critical for the development and function of the central nervous system (CNS).

In the present study, we leveraged the ability to prevent the transport of lysosome-related vesicles into the axon by KO of BORC subunits to examine the impact of this manipulation on the repertoire of mRNAs, the transport of specific mRNAs and the impact on other organelles in the axon. We found that KO of BORC subunits in human neurons derived from induced pluripotent stem cells (iPSCs) led to a substantial reduction in lysosome-related vesicles and a depletion of a large subset of mRNAs in the axon. Most prominent among the depleted mRNAs were those encoding components of ribosomes and of the mitochondrial oxidative phosphorylation system (OXPHOS). This resulted in decreased protein translation and mitochondrial abnormalities. Moreover, the depleted mRNAs were associated with pathways of neurodegeneration in Parkinson’s, Alzheimer’s, Huntington’s and prion diseases and in amyotrophic lateral sclerosis (ALS). These findings indicate that hitchhiking on lysosome-related vesicles is a major pathway for the transport of mRNAs into the axon and that this process is critical for the maintenance of mitochondrial homeostasis. In addition, our findings reveal a plausible mechanism for axonal degeneration in humans with mutations in BORC subunits that is related to the pathogenesis of common neurodegenerative disorders.

## Results

### BORC-KO depletes axonal lysosome-related vesicles

To investigate the role of axonal transport of lysosome-related vesicles in mRNA transport and axonal homeostasis, we used human iPSCs expressing the neuronal transcriptional activator neurogenin 2 (NGN2) under the control of a doxycycline-inducible promoter^36,37^. Culture of these iPSCs in doxycycline-containing medium induces their differentiation into cortical-like, glutamatergic neurons (termed i3Neurons)^36,37^ (Fig. 1b). We used CRISPR–Cas9 to KO the genes encoding the BORCS5 (also known as myrlysin) or BORCS7 (also known as lyspersin) subunit of BORC^31^ in these iPSCs and stable lentiviral re-expression of BORCS5 or BORCS7, respectively, in the KO iPSCs (Fig. 1c). Immunofluorescence microscopy of wild-type (WT) i3Neurons cultured on glass coverslips for 25 d showed that they had distinct soma and dendrites labeled for the somatodendritic marker MAP2 and axons labeled for the synaptic vesicle marker SYP (Fig. 1d; WT). The axonal domain was extensively developed at this time, appearing as a lace-like network on the coverslips. Lysosomal vesicles were most concentrated in the soma, but they were also apparent as fine puncta in the dendrites and axons, as detected by staining for the lysosomal proteins LAMP1 or LAMTOR4 (Fig. 1e; WT).

**Extended Data Fig. 1:**
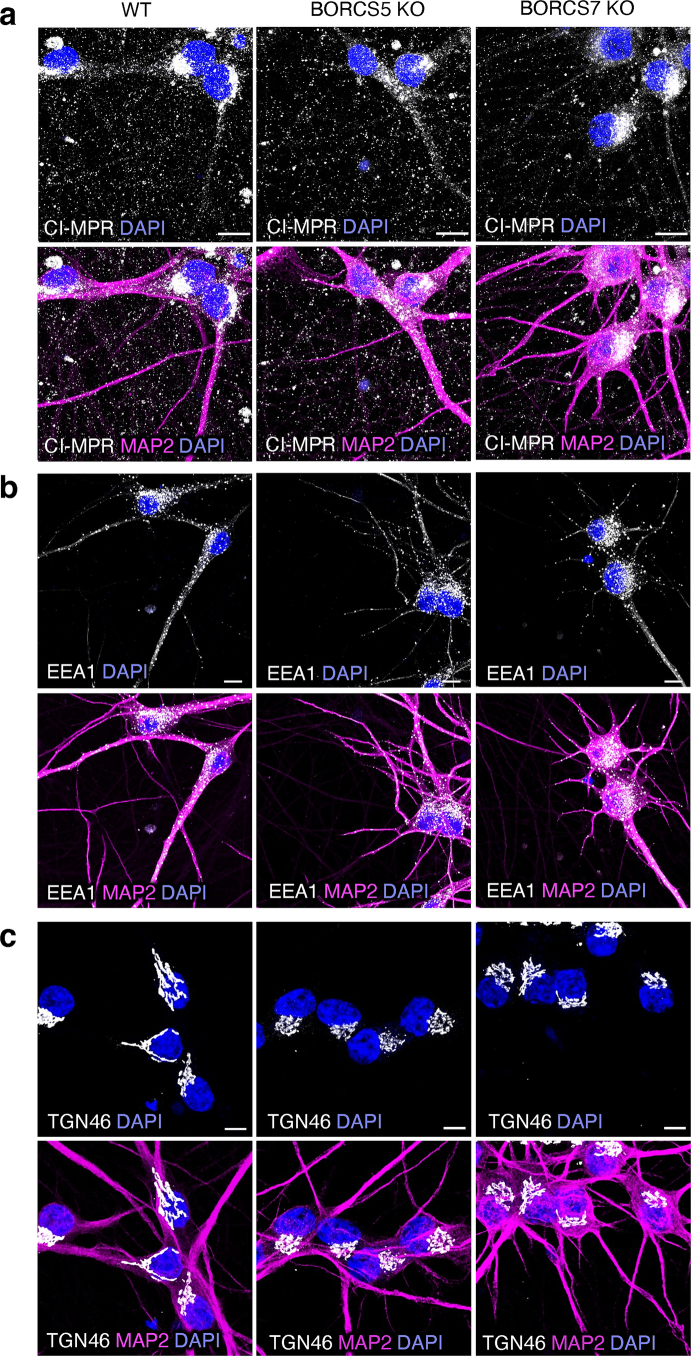
Distribution of endosomal and TGN markers is not affected in BORC-KO i3Neurons. **a-c**, WT, BORCS5-KO and BORCS7-KO i3Neurons grown for 25 days on glass coverslips were fixed, permeabilized and co-stained with antibodies to endogenous MAP2 (soma and dendrites) (magenta) (all three panels) and cation-independent mannose 6-phosphate receptor (CI-MPR) (endosomes and TGN) (**a**), early endosomal antigen 1 (EEA1) (early endosomes) (**b**), or TGN46 (TGN) (**c**) (all in grayscale). Nuclear DNA was stained with DAPI (blue). Scale bars: 10 μm. Notice the presence of CI-MPR in all neuronal domains including axons (**a**), EEA1 only in the soma and dendrites (**b**) and TGN46 only in the soma (**c**), and how the distribution of all these markers does not change upon BORCS5 KO or BORCS7 KO. All the experiments shown in **a-c** were repeated three times.

**Extended Data Fig. 2:**
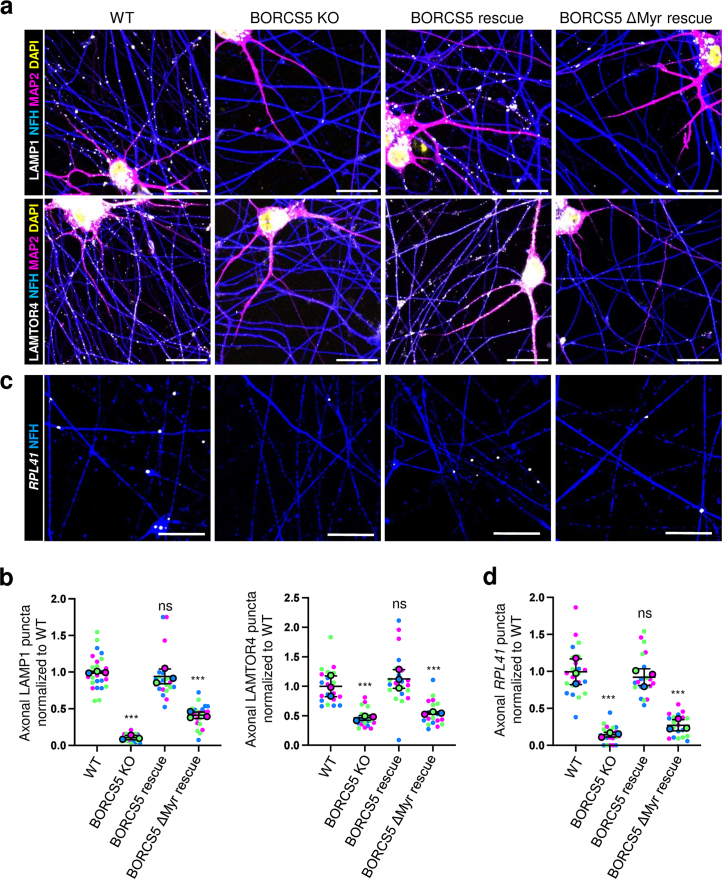
Decreased *RPL41* mRNA staining in axons from BORCS5-KO i3Neurons. **a**, i3Neurons [WT, BORCS5 KO, BORCS5 KO rescued with WT BORCS5, and BORCS5 KO rescued with a G2A, myristoylation-defective BORCS5 (ΔMyr)] were immunostained for endogenous MAP2 (soma and dendrites) (magenta), neurofilament heavy chain (NFH) (axons, blue), LAMP1 (lysosomes) or LAMTOR4 (lysosomes) (all grayscale). Nuclei were stained with DAPI (yellow). Scale bars: 20 μm. Experiments were repeated three times. **b**, Quantification of the number of axonal puncta staining for LAMP1 or LAMTOR4 in the indicated i3Neuron lines relative to WT i3Neurons (defined as 1.0) from three experiments such as that shown in panel **a**. Results are represented as SuperPlots, showing the individual data points and mean of the means ± SD. Statistical significance was calculated by one-way ANOVA with Dunnett’s multiple comparisons test. Significance relative to WT: LAMP1, BORCS5 KO ***P < 0.001, BORCS5 rescue *P* = 0.586, BORCS5 ΔMyr rescue ***P < 0.001. LAMTOR4, BORCS5 KO ***P < 0.001, BORCS5 rescue *P* = 0.382, BORCS5 ΔMyr rescue ***P < 0.001. ns: not significant. Notice the depletion of lysosome-related vesicles from the axon of BORCS5-KO neurons and the rescue of this phenotype by WT but not ΔMyr BORCS5. **c**, The same i3Neuron lines shown in panel **a** were grown on coverslips for 20 days, fixed and processed for RNAscope in situ hybridization using a probe for *RPL41* mRNA (white dots). Cells were also immunostained for NFH (axons, blue). Scale bars: 20 μm. Experiments were repeated three times. **d**, Quantification of the number of *RPL41* dots per axon unit area in the indicated i3Neuron lines relative to WT i3Neurons (defined as 1.0) from three experiments such as that shown in panel **c**. Results are represented as SuperPlots, showing the individual data points and mean of the means ± SD. Statistical significance was calculated by one-way ANOVA with Dunnett’s multiple comparisons test. Significance relative to WT: *RPL41*, BORCS5 KO ***P < 0.001, BORCS5 rescue *P* = 0.780, BORCS5 ΔMyr rescue ***P < 0.001. Notice the depletion of *RPL41* mRNA in the axon of BORCS5-KO neurons, and the rescue of this phenotype by WT but not ΔMyr BORCS5, paralleling the results for lysosome-related vesicles.

BORCS5 or BORCS7 KO (referred to as BORC-KO) did not prevent the differentiation of the iPSCs into i3Neurons. Additionally, the neurons had well-defined somatodendritic and axonal domains (Fig. 1d). Furthermore, these KOs had no effect on the presence of synaptic vesicles labeled for SYP and mitochondria labeled for TOMM20 in the axon (Fig. 1d). Also unaffected were the distributions of TGN or endosomal markers, such as CI-MPR, EEA1 and TGN46 (Extended Data Fig. 1). In contrast, BORCS5 or BORCS7 KO caused a marked depletion of lysosome-related vesicles from the axon (Fig. 1e,f and Extended Data Fig. 2a,b). The presence of axons in the fields devoid of LAMP1 was evidenced by SYP and TOMM20 staining (Fig. 1d) and by increasing the brightness of the MAP2 channel (Fig. 1e; MAP2 bright). Stable re-expression of BORCS5 or BORCS7 in the corresponding KO cells restored the presence of lysosome-related vesicles in the axon (Fig. 1e,f and Extended Data Fig. 2a,b). These experiments thus demonstrated that BORC-KO specifically depleted lysosome-related vesicles from the axon of human i3Neurons, as previously shown for silencing of BORC subunits in rat and mouse hippocampal and cortical neurons^6,7,11^.

### Axonal enrichment of ribosomal and mitochondrial protein mRNAs

**Fig. 2:**
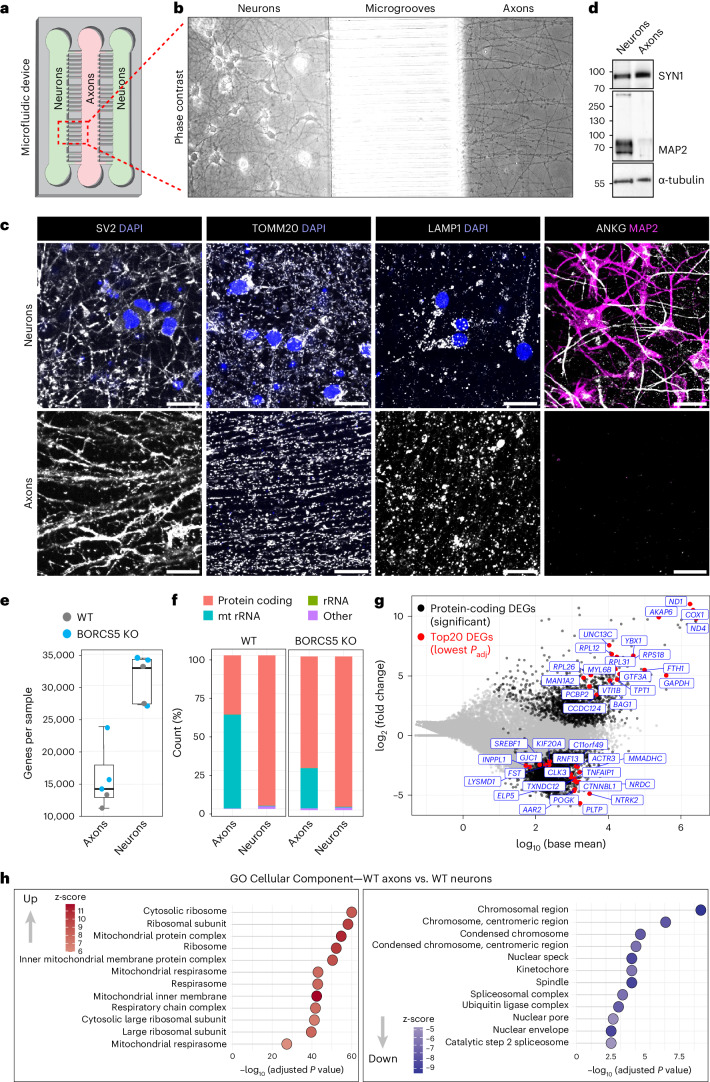
Axonal mRNA profile of i3Neurons cultured in a microfluidic device. **a**, Schematic representation of a microfluidic device designed to isolate pure axons. The device is composed of three chambers separated by two sets of microgrooves. i3Neurons were plated on the two side chambers (green) and cultured for 45 d, during which time axons grew toward the central chamber (pink). **b**, Phase-contrast image of neurons and axons grown in the microfluidic device. **c**, i3Neurons grown in the microfluidic device were immunostained for the indicated proteins (all grayscale and/or magenta). Nuclei were stained with DAPI (blue). The experiment was repeated three times. Scale bars, 20 μm. **d**, Immunoblot analysis of protein extracts from neuronal and axonal compartments of the microfluidic device. The immunoblot was repeated two times. The positions of molecular mass markers (in kDa) are indicated on the left. **e**, RNA extracted from the axonal and neuronal compartments of *n* = 2 WT and *n* = 3 BORCS5 KO independent cultures of i3Neurons was subjected to RNA-seq. The number of genes with non-zero read counts was counted without normalization across the biological replicates. Box plots show the individual data points, median (center line indicating the 50th percentile), 75th percentile (top of the box) and 25th percentile (bottom of the box). The whiskers extend to the maximum and minimum data value that is no more than 1.5 times the interquartile range above or below the hinge. **f**, Proportion of the indicated RNA biotypes in axonal and neuronal preparations. **g**, MA plot of protein-coding genes in WT axons compared to WT neurons. Each dot represents a protein-coding gene with its mean normalized read count in log_10_ scale (*x* axis) and fold change in log_2_ scale (*y* axis). Insignificant (FDR > 0.1), significant (FDR < 0.1) and top 20 (lowest FDR) protein-coding DEGs for both up or down DEGs are colored gray, black and red, respectively. Top 20 gene names are indicated. **h**, Dot plots for GO Cellular Component gene sets enriched in WT axons versus WT neurons in RNA-seq. Enriched gene sets were arranged by statistical significance (FDR). The z-score captures both the direction of changes and the number of genes changing in each direction. A larger absolute z-score indicates a more biased direction toward increase or decrease. Statistical significance was calculated by one-sided Fisher’s exact test. *P* values were adjusted for multiple comparisons using the Benjamini–Hochberg method. Source data

**Extended Data Fig. 3:**
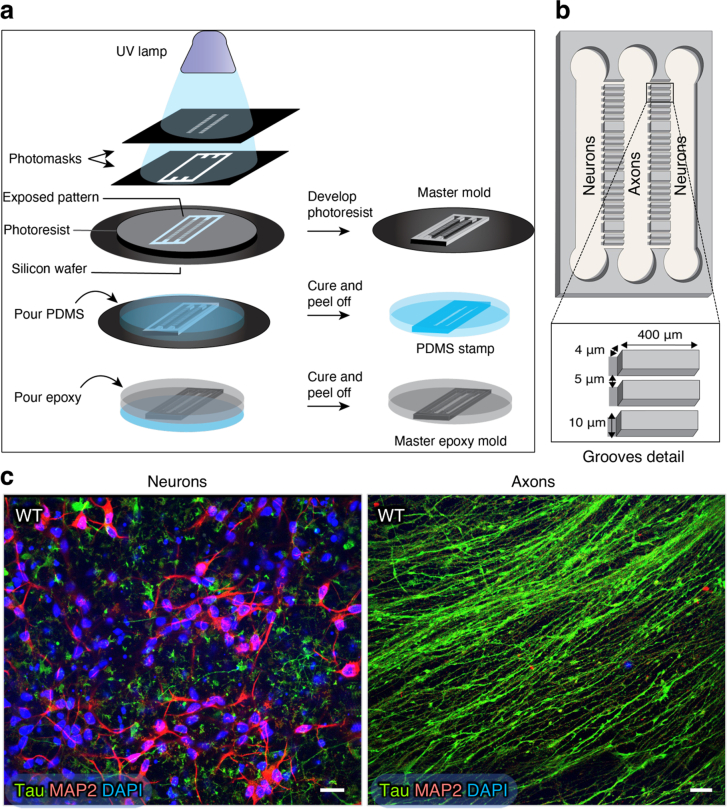
Design of a microfluidic device for isolation of axons from i3Neurons. **a**, Schematic representation of the method used to prepare a microfluidic device. PDMS: polydimethylsiloxane. **b**, The device is composed of three open chambers approximately 2-mm wide by 40-mm long, separated by two sets of ~2,400 microgrooves with dimensions shown. A slightly wider gap (115 mm) between microgrooves is used every 1.7 mm to improve stability of the mounted device (see Supplementary Data 2 and 3 for CAD files of mask design). The PDMS component is represented in gray. Pre-differentiated i3Neurons are plated on the lateral chambers and axons grow into the middle chamber. This device provides a substantially larger culture area for neuronal seeding (400 mm^2^ in the two outer chambers combined) and for axonal growth (200 mm^2^ in the central chamber) compared to available commercial options. The open-chamber design provides an improved culture environment for the cells because it facilitates gas exchange with the medium and offers easier access for initial seeding, reagent addition, and sample collection. The high density of the microgrooves (60 microgrooves/mm), the ability to seed neurons close to the microgrooves, and the proximity of the entire area of the axon chamber to the microgrooves all help to increase the isolation throughput. **c**, WT i3Neurons grown for 45 days in a microfluidic device were immunostained for endogenous Tau (axons) and MAP2 (soma and dendrites). Nuclei were stained with DAPI. Images were obtained by confocal fluorescence microscopy. Representative images from the neuronal (left) or axonal (right) compartments are shown. Experiments were repeated three times. Notice the absence of somatodendritic and nuclear markers in the axonal compartment, indicating the purity of the axonal preparation. Scale bars: 20 μm.

To analyze the mRNA profile in the axon of WT and BORC-KO i3Neurons, we built a microfluidic device that allows isolation of sufficient axonal material for transcriptomic analyses (Fig. 2a,b and Extended Data Fig. 3a,b). The iPSCs were plated on the two side chambers, and, upon differentiation into i3Neurons, only axons were thin and long enough to grow into the middle chamber (Fig. 2a,b). Immunofluorescence microscopy of WT i3Neurons grown for 45 d showed that the middle chamber contained numerous axons staining for the microtubule-associated protein Tau (Extended Data Fig. 3c), the synaptic vesicle protein SV2, the mitochondrial protein TOMM20 and the lysosomal protein LAMP1 but not the somatodendritic marker MAP2 and the axon initial segment (AIS) protein ankyrin G (ANKG) (Fig. 2c and Extended Data Fig. 3c). The side chambers stained for all these markers as well as the nuclear DNA dye DAPI (Fig. 2c and Extended Data Fig. 3c). Therefore, the middle chamber contained pure axons, whereas the two side chambers contained all neuronal domains, including axons and the AIS. On this basis, we refer to preparations from these chambers as ‘axons’ and ‘neurons’, respectively. Immunoblot analysis of these preparations confirmed enrichment of the synaptic vesicle protein SYN1 and depletion of MAP2 relative in the axon relative to the neurons (Fig. 2d).

We performed RNA sequencing (RNA-seq) and differential expression analysis in axon and neuron preparations from day-45 i3Neurons (Fig. 2e–h and Supplementary Tables 1 and 2). These analyses identified transcripts from an average of 11,089 axonal and 29,410 neuronal genes in WT and 16,867 axonal and 31,211 neuronal genes in BORCS5-KO i3Neurons (Fig. 2e). In WT axons, 61% of the RNA counts corresponded to mitochondrial ribosomal RNAs (mt rRNAs) and 38% to protein-coding mRNAs (Fig. 2f). In WT neurons, on the other hand, over 98% of the RNAs were protein coding (Fig. 2f). The abundance of mt rRNAs in the axon was previously reported for embryonic mouse and human motor neurons and likely reflects the enrichment of mitochondria in axons^38–41^. A large subset of protein-coding transcripts (1,628 genes) was significantly more abundant in WT axons relative to WT neurons (Fig. 2g). Among the most enriched transcripts in WT axons were mitochondria-encoded mRNAs for OXPHOS proteins (ND1, ND4 and COX1) and nuclear-encoded mRNAs for ribosomal proteins (RPL12, RPL26, RPL31 and RPS18) (Fig. 2g and Supplementary Tables 1 and 2). Gene Ontology (GO) Cellular Component analysis of all axon-enriched transcripts in WT neurons showed enrichment in mRNAs for ribosomal and mitochondrial proteins, both nuclear encoded and mitochondria encoded (Fig. 2h), as previously reported for other axonal or nerve terminal preparations^38,40–44^. In contrast, the neuron fraction was most enriched in mRNAs coding for chromosomal and nuclear proteins (Fig. 2h).

### BORC-KO depletes axonal mRNAs for ribosomal and OXPHOS proteins

**Fig. 3:**
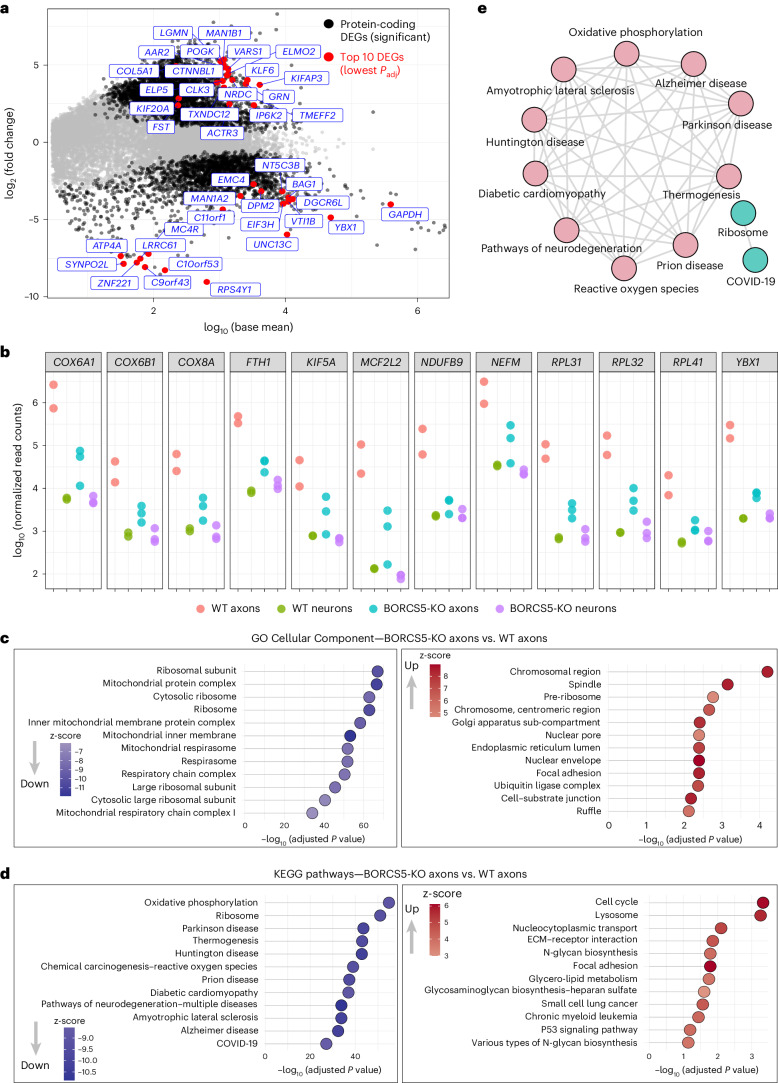
Depletion of mRNAs encoding ribosomal and mitochondrial proteins from the axon of BORCS5-KO i3Neurons. **a**, MA plot for protein-coding genes in BORCS5-KO axons versus WT axons identified by RNA-seq. Each dot represents a protein-coding gene with its mean normalized read count (*x* axis) and log_2_ fold change (*y* axis). Insignificant (FDR > 0.1), significant (FDR < 0.1) and top 20 (lowest FDR) protein-coding DEGs for both up or down DEGs are colored gray, black and red, respectively. Top 20 up or down genes are indicated. **b**, Expression profiling of selected axonal genes shown to be axon enriched in i3Neurons (the present study) and other neuronal types^38,40–42^. Each dot represents a biological replicate, with log_10_-normalized read counts for each gene on the *y* axis. **c**,**d**, Dot plots for gene sets in GO Cellular Component (**c**) and KEGG pathways (**d**). Left panel (down) represents the set of genes that had a negative interaction term in the axon and no change in neurons due to BORCS5 KO; right panel (up) shows the set of genes that had a positive interaction term in the axon and no change in neurons due to BORCS5 KO. Enriched gene sets in **c** and **d** were arranged by statistical significance (FDR). The z-score captures both the direction of changes and the number of genes changing in each direction. A larger absolute z-score indicates a more biased direction toward up or down. Statistical significance was calculated by one-sided Fisher’s exact test. *P* values were adjusted for multiple comparisons using the Benjamini–Hochberg method. **e**, Enrichment map for top 12 KEGG gene sets decreased in BORCS5-KO axons versus WT axons in RNA-seq. The gene sets in the left panel of **d** were clustered by a community detection algorithm. The map consists of nodes for pathways and edges indicating the presence of DEGs that are concurrently found between the pathways.

Next, we evaluated the impact of depleting axonal lysosome-related vesicles on the repertoire of axonal mRNAs. We observed a decrease in the proportion of axonal mt rRNAs in BORCS5 KO (26%) relative to WT i3Neurons (61%) (Fig. 2f). Comparison of specific protein-coding mRNAs in WT versus BORCS5-KO axons revealed that BORCS5 KO resulted in a marked decrease in a subset of mRNAs (2,230 genes) (Fig. 3a). Many of these mRNAs corresponded to those that are normally enriched in the axon (Fig. 2g and Supplementary Tables 1 and 2)^38–42^, as exemplified by those shown in Fig. 3b.

**Extended Data Fig. 4:**
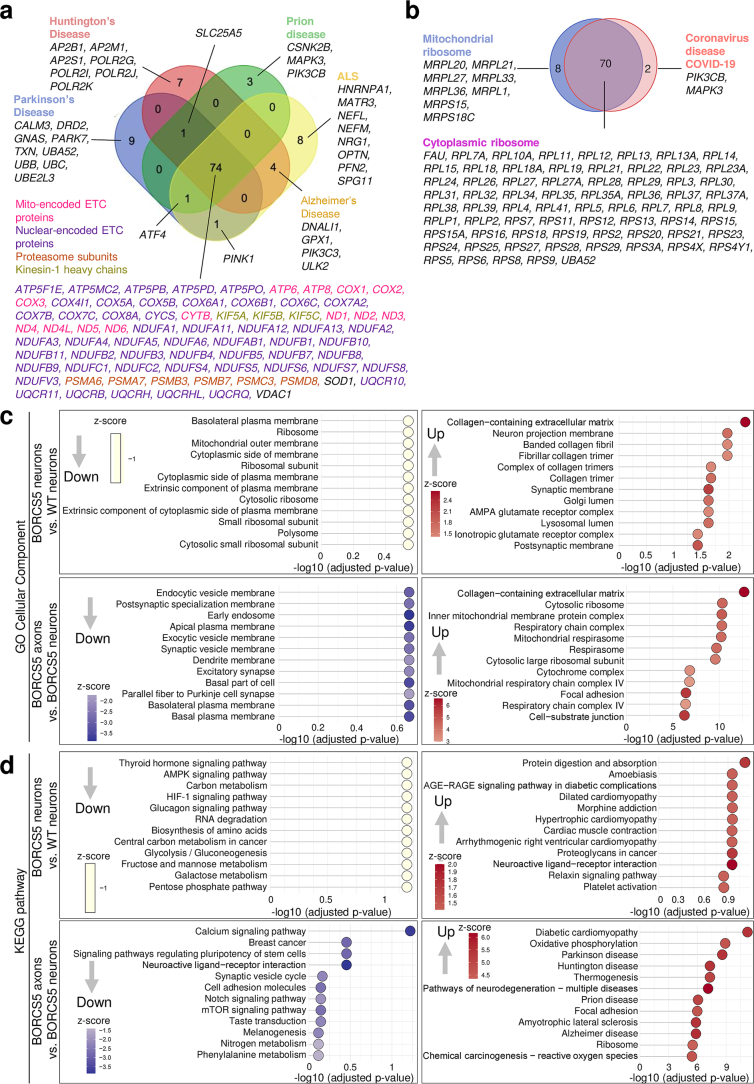
Relationship of mRNA sets depleted in BORCS5-KO axons to neurodegenerative disorders and COVID-19, and GO Cellular Component and KEGG pathway analysis of changed mRNA sets. **a**, Venn diagram for functional enrichment related to neurodegenerative disorders. DEGs that were down in BORCS5-KO axons compared to WT axons matched to gene sets related to neurodegenerative disorders, as defined by KEGG. **b**, Venn diagram for functional enrichment related to ribosomes and COVID-19. DEGs that were down in BORCS5-KO axons compared to WT axons matched to gene sets related to ribosome and COVID-19, as defined by KEGG. **c**, GO Cellular Component analysis of gene sets that are increased or decreased in BORCS5-KO neurons vs WT neurons and BORCS5-KO axons vs BORCS5-KO neurons. **d**, KEGG pathway analysis of gene sets that are increased or decreased in BORCS5-KO neurons vs WT neurons and BORCS5-KO axons vs BORCS5-KO neurons. Both **c** and **d** show dot plots for DEG sets that are increased (up) or decreased (down) in RNA-Seq. Enriched terms are arranged by statistical significance (FDR), showing the top 12 terms. The z-score captures both the direction of changes and the number of genes changing in each direction. A larger absolute z-score indicates a more biased direction towards increase or decrease. Statistical significance was calculated by one-sided Fisher’s exact test. *P*-values were adjusted for multiple comparisons using the Benjamini-Hochberg method.

GO Cellular Component and KEGG functional enrichment analyses showed that the most significant terms of axonal mRNAs depleted in BORCS5-KO i3Neurons corresponded to those encoding ribosomal and mitochondrial proteins (Fig. 3c,d). KEGG also showed that 6 of the top 12 terms corresponded to neurodegenerative disorders (Parkinson’s disease, Huntington’s disease, prion disease, pathways of neurodegeneration–multiple diseases, ALS and Alzheimer’s disease) (Fig. 3d), suggesting a common mechanism of axonal pathology in BORC-deficient neurons and neurodegenerative disorders. All these disorders, as well as diabetic cardiomyopathy, thermogenesis and reactive oxygen species (ROS), share a relationship to OXPHOS (Fig. 3d,e). The mRNAs depleted in BORCS5-KO axons and related to common neurodegenerative disorders include both nuclear-encoded and mitochondria-encoded mRNAs for components of electron transport chain complexes, proteasome subunits and kinesin-1 heavy chains (Extended Data Fig. 4a and Supplementary Table 2). They also include mRNAs for proteins that are mutated in neurodegenerative disorders (Extended Data Fig. 4a and Supplementary Table 2). The ribosome category, on the other hand, shows a relationship to coronavirus disease 2019 (COVID-19) (Fig. 3e, Extended Data Fig. 4b and Supplementary Table 2). This category includes transcripts for both cytoplasmic and mitochondrial ribosomes, with only the former relating to COVID-19 (Extended Data Fig. 4b and Supplementary Table 2). These results are consistent with a defect in the transport of specific populations of nuclear-encoded mRNAs into the axon and with a reduction in transcription or stability of mitochondria-encoded mRNAs.

BORCS5 KO also caused a relative increase in a different subset of mRNAs (3,533 genes) (Fig. 3a). In contrast to the predominance of a few GO categories, such as mitochondria and ribosomes among the depleted mRNAs in BORCS5-KO i3Neurons, the increased mRNAs belonged to diverse categories, including chromosomes, mitotic spindle, endoplasmic reticulum, nuclear envelope and focal adhesion (Fig. 3c), which also correspond to mRNAs that are abundant in whole neurons (Fig. 2h). Notably, the second-most increased mRNA term in the KEGG analysis was lysosome (Fig. 3d), perhaps reflecting a compensatory mechanism to generate lysosome-related vesicles when their transport is impaired. Thus, BORCS5 KO decreases axon-enriched mRNAs while increasing neuron-enriched mRNAs in the axon.

The comparison of BORCS5-KO versus WT neurons showed two orders of magnitude fewer genes detected as differentially expressed relative to BORCS5-KO versus WT axons (Supplementary Table 1). Furthermore, KEGG and GO analyses showed little difference in the mRNA profiles of BORCS5 KO versus WT neurons (Extended Data Fig. 4c,d). These observations indicated that the impact of BORCS5 KO was most pronounced in the mRNA profile of axons.

Using RNAscope in situ hybridization (ISH), we observed a reduction in the signal for *RPL41* mRNA in the axon of BORCS5-KO i3Neurons and restoration to normal levels upon re-expression of BORCS5 (Extended Data Fig. 2c,d), confirming the results from RNA-seq (Supplementary Tables 1 and 2). Substitution of alanine for glycine-2, which prevents myristoylation of BORCS5 and its recruitment to lysosomes^31^, greatly reduced the ability of BORCS5 to rescue the localization of both lysosome-related vesicles and *RPL41* mRNA (Extended Data Fig. 2a–d).

### BORC-KO decreases axonal transport of mRNAs for ribosomal proteins

**Fig. 4:**
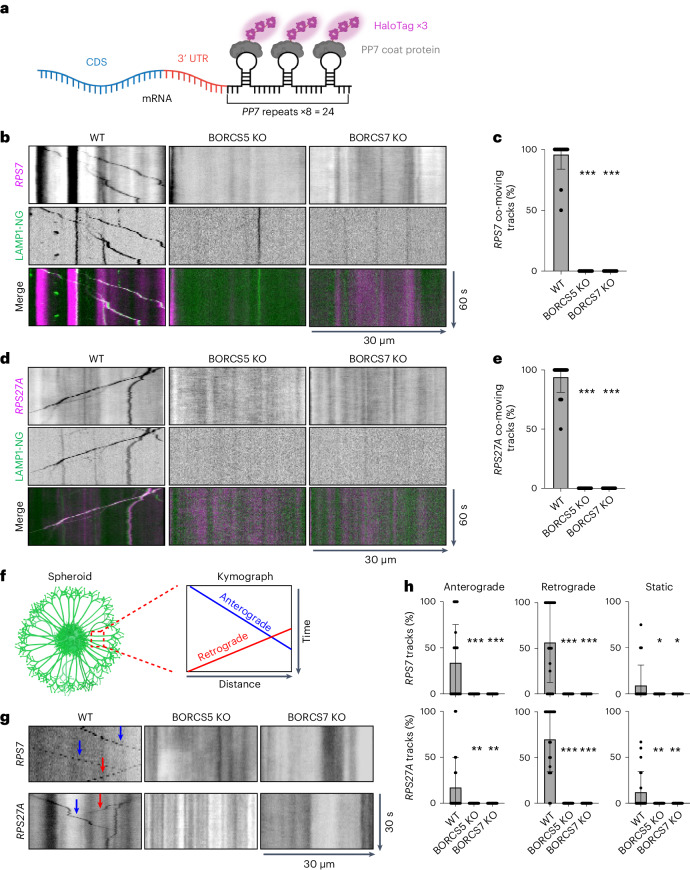
Transport of mRNAs encoding ribosomal proteins is reduced in the axon of BORC-subunit-KO i3Neurons. **a**, Constructs used for RNA visualization. A construct encoding HaloTags fused to the PP7 coat protein was stably co-expressed with the coding sequencing (CDS) of the gene of interest followed by its 3′ UTR, fused to PP7 RNA stem-loop repeats in WT, BORCS5-KO or BORCS7-KO i3Neurons. **b**,**d**, Kymographs of *RPS7* (**b**) or *RPS27A* (**d**) i3Neurons co-expressing the constructs described in **a** were transduced with LAMP1-mNeonGreen (LAMP1-NG) and incubated with the fluorescent Halo substrate JF646 to image axonal lysosome-related vesicle and mRNA movement, respectively. Axons were imaged live for 60 s. Kymographs were generated from Supplementary Videos 1 and 2. Single-color images are represented in inverted grayscale. See Extended Data Fig. 6 for negative control of JF646 staining. **c**,**e**, Quantification of co-moving LAMP1-NG and *RPS7* (**c**) or *RPS27A* (**e**) mRNA tracks. Values are the mean ± s.d. of ~22 kymographs from ~22 neurons per condition and are expressed as the percentage of the indicated marker that co-moves with LAMP1-NG. Statistical significance was calculated by one-way ANOVA with Dunnett’s multiple comparisons test. BORCS5 KO versus WT ***P < 0.001 and BORCS7 KO versus WT ***P < 0.001 for both **c** and **e**. **f**, Scheme of a neuronal spheroid. Axons were imaged live for 30 s, and kymographs were generated from the videos. Lines with negative or positive slopes represent anterograde or retrograde movement, respectively. **g**, Kymographs of axonal mRNA movement in i3Neurons co-expressing combinations of *RPS7*-PP7 or *RPS27A*-PP7 repeats and HaloTag-PP7 coat constructs were generated from spheroids. **h**, Quantification of anterograde, retrograde and static *RPS7* or *RPS27A* mRNA tracks. Values are the mean ± s.d. from ~20 kymographs from ~20 neurons per condition and are expressed as the percentage of the indicated marker in anterograde, retrograde or static particles. Statistical significance was calculated by one-way ANOVA with Dunnett’s multiple comparisons test. *P* values relative to WT: *RPS7* anterograde, BORCS5 KO ***P < 0.001, BORCS7 KO ***P < 0.001; retrograde, BORCS5 KO ***P < 0.001, BORCS7 KO ***P < 0.001; static, BORCS5 KO *P = 0.023, BORCS7 KO *P = 0.023. *RPS27A* anterograde, BORCS5 KO **P = 0.004, BORCS7 KO **P = 0.004; retrograde, BORCS5 KO ***P < 0.001, BORCS7 KO ***P < 0.001; static, BORCS5 KO **P = 0.003, BORCS7 KO **P = 0.003.

**Extended Data Fig. 5:**
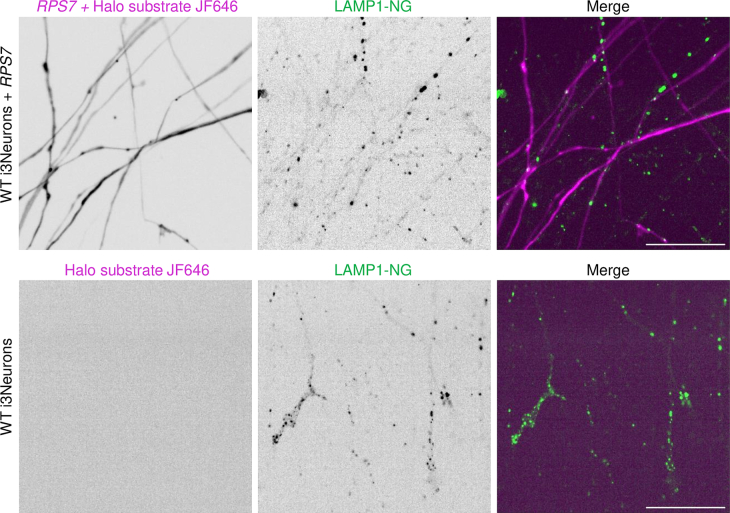
Control experiment demonstrating the specificity of HaloTag staining in mRNA transport experiments. WT i3Neurons co-expressing 3 HaloTags fused to PP7 coat protein and *RPS7* fused to 24 PP7 RNA stem-loop repeats (top row) (see scheme in Fig. 4a), or untransfected WT i3Neurons (negative control) (bottom row), were grown on coverslips for 25 days and transduced with LAMP1-mNeonGreen (LAMP1-NG) for 36 h. Neurons were then incubated overnight with 200 pM of the fluorescent HaloTag substrate JF646 and imaged by spinning-disk confocal microscopy. Scale bars: 20 μm. Notice the absence of JF646 signal in the control i3Neurons, demonstrating that the JF646 signal represents *RPS7* staining and not non-specific staining of lysosome-related vesicles or other vesicles. Experiments were repeated three times.

To directly observe if the depletion of specific mRNAs from the axon of BORCS5-KO neurons was due to decreased transport, we examined the axonal transport of *RPS7* and *RPS27A*, both of which were significantly reduced in BORCS5-KO relative to WT i3Neurons (Supplementary Tables 1 and 2). This analysis was done by stable co-expression of constructs encoding 24 *PP7* RNA stem-loop repeats fused to the mRNA of interest and the PP7 coat protein fused to a triple HaloTag (Fig. 4a)^45^. To visualize the movement of mRNA and lysosome-related vesicles, we plated the co-transfected WT i3Neurons on coverslips and infected them with a lentivirus encoding LAMP1-mNeonGreen. We then incubated the cells overnight with 200 pM of the Halo substrate JF646. As a control, we showed that untransfected WT i3Neurons displayed no Halo signal (Extended Data Fig. 5). Live-cell imaging showed co-movement of LAMP1-mNeonGreen with both *RPS7* and *RPS27A* in WT axons (Fig. 4b–e and Supplementary Videos 1 and 2) and no movement of any of these molecules in BORCS5-KO or BORCS7-KO axons (Fig. 4b–e and Supplementary Data 4).

To determine transport direction, we turned to a system in which WT, BORCS5-KO and BORCS7-KO i3Neurons were grown as three-dimensional (3D) spheroids^46^, with axons extending outward (Fig. 4f). In WT axons, we observed both anterograde and retrograde trajectories, as well as a few static foci, labeled for *RPS7* and *RPS27A* (Fig. 4g,h and Supplementary Data 4). In BORCS5-KO and BORCS7-KO axons, we could not detect any moving *RPS7* and *RPS27A* (Fig. 4g,h and Supplementary Data 4). These experiments demonstrated that BORCS5 and BORCS7, and, therefore, transport of lysosome-related vesicles, are required for *RPS7* and *RPS27A* transport within the axon. The absence of retrogradely moving mRNAs is likely secondary to the inhibition of anterograde mRNA transport in the BORCS5-KO and BORCS7-KO neurons.

**Extended Data Fig. 6:**
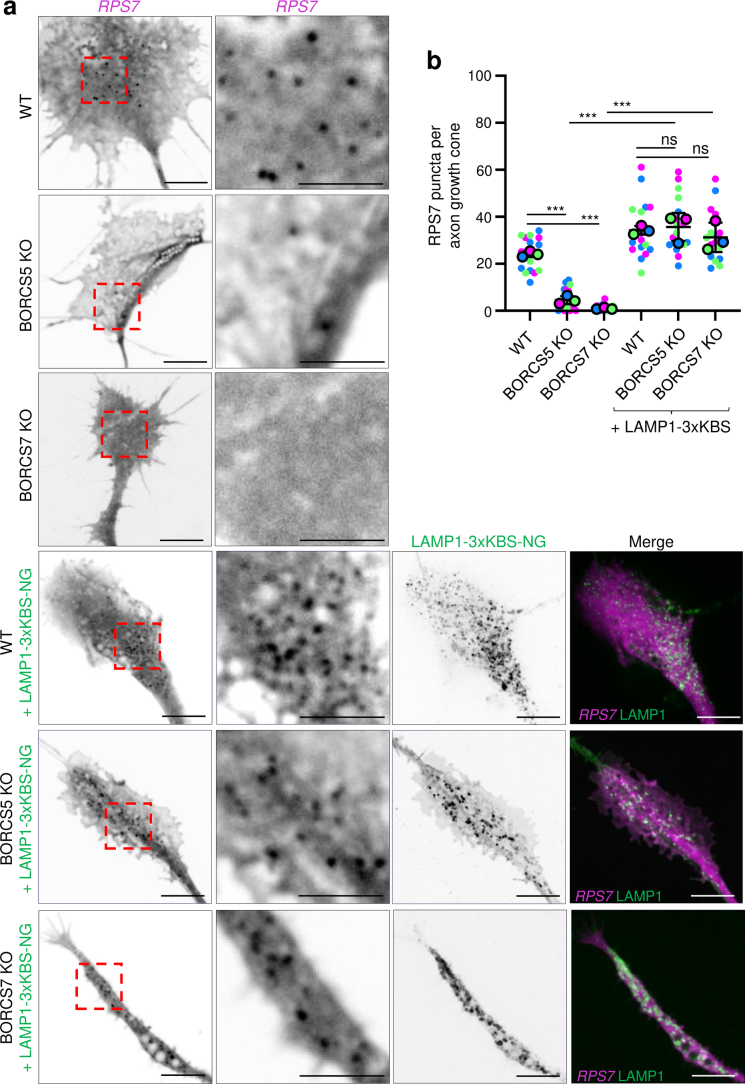
BORC-independent rescue of axonal *RPS7* mRNA by expression of LAMP1-3xKBS in BORCS5-KO and BORCS7-KO i3Neurons. **a**, WT, BORCS5-KO and BORCS7-KO i3Neurons co-expressing the PP7 coat protein fused with three HaloTags and *RPS7* CDS fused with 24 PP7 RNA stem-loop repeats were grown on coverslips for 15 days. Cells were then transduced with a construct encoding three repeats of a kinesin-binding sequence (KBS) fused to NeonGreen-tagged LAMP1 (LAMP1-3xKBS-NG) for 36 h and incubated with 200 pM of the fluorescent Halo substrate JF646 overnight to image lysosome-related vesicles and *RPS7* mRNA localization at axon terminals. Scale bars for left, LAMP1-3xKBS-NG and merged columns: 10 μm. Scale bars for *RPS7* zoomed-in boxed areas: 5 μm. **b**, Quantification of the number of *RPS7* puncta per axon terminal from n = 3 independent experiments. Values are represented as SuperPlots, showing the individual data points and the mean of the means ± SD. Statistical significance was calculated by one-way ANOVA with Dunnett’s multiple comparisons test. BORCS5 KO vs WT ***P < 0.001, BORCS7 KO vs WT ***P < 0.001, BORCS5 KO + LAMP1-3xKBS vs BORCS5 KO ***P < 0.001, BORCS7 KO + LAMP1-3xKBS vs BORCS7 KO ***P < 0.001, BORCS5 KO + LAMP1-3xKBS vs WT + LAMP1-3xKBS *P* = 0.998, BORCS7 KO + LAMP1-3xKBS vs WT + LAMP1-3xKBS *P* = 0.949. ns: not significant relative to WT. Notice the increased number of *RPS7* mRNA and lysosomal puncta in BORCS5-KO and BORCS7-KO i3Neurons transduced with LAMP1-3xKBS-NG.

**Extended Data Fig. 7:**
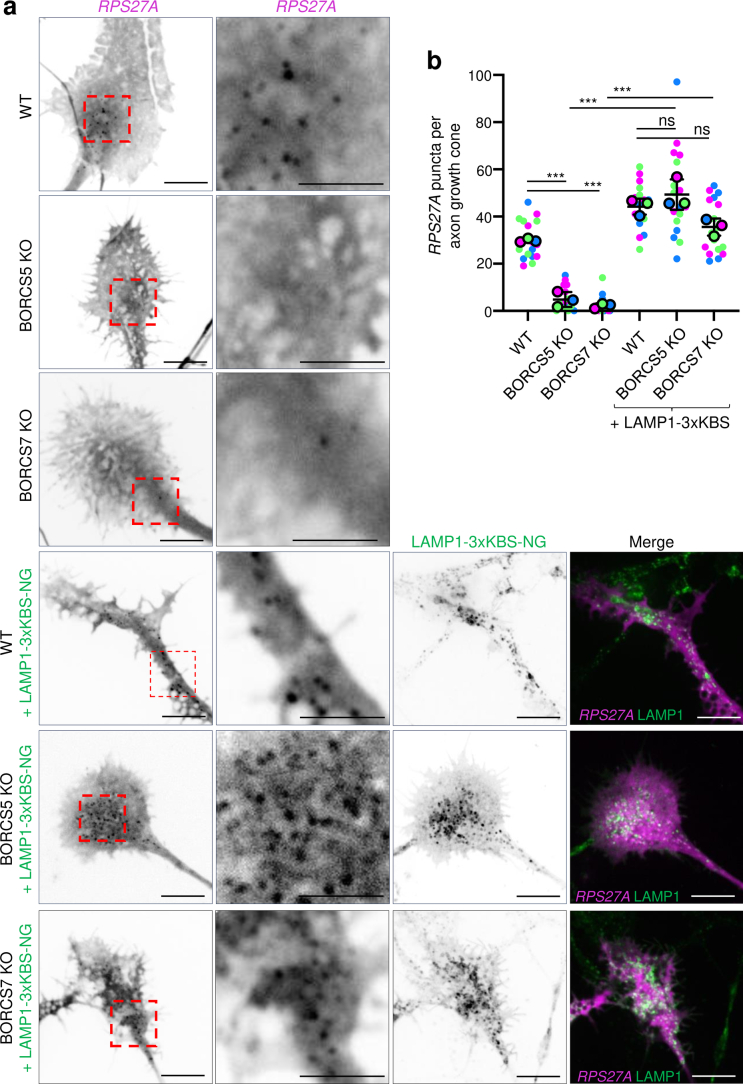
BORC-independent rescue of axonal *RPS27A* mRNA by expression of LAMP1-3xKBS in BORCS5-KO and BORCS7-KO i3Neurons. **a**, WT, BORCS5-KO and BORCS7-KO i3Neurons co-expressing the PP7 coat protein fused with three HaloTags and *RPS27A* CDS fused with 24 PP7 RNA stem-loop repeats were grown on coverslips for 15 days. Cells were then transduced with a construct encoding LAMP1-3xKBS-NG for 36 h and incubated with 200 pM of the fluorescent Halo substrate JF646 overnight to image lysosome-related vesicles and *RPS27A* mRNA localization at axon terminals. Scale bars for left, LAMP1-3xKBS-NG and merged columns: 10 μm. Scale bars for *RPS27A* zoomed-in boxed areas: 5 μm. **b**, Quantification of the number of *RPS27A* puncta per axon terminal from n = 3 independent experiments. Values are represented as SuperPlots, showing the individual data points and the mean of the means ± SD. Statistical significance was calculated by one-way ANOVA with Dunnett’s multiple comparisons test. BORCS5 KO vs WT ***P < 0.001, BORCS7 KO vs WT ***P < 0.001, BORCS5 KO + LAMP1-3xKBS vs BORCS5 KO ***P < 0.001, BORCS7 KO + LAMP1-3xKBS vs BORCS7 KO ***P < 0.001, BORCS5 KO + LAMP1-3xKBS vs WT + LAMP1-3xKBS *P* = 0.721, BORCS7 KO + LAMP1-3xKBS vs WT + LAMP1-3xKBS *P* = 0.136. ns: not significant relative to WT. Notice the increased number of *RPS27A* mRNA and lysosomal puncta in BORCS5-KO and BORCS7-KO i3Neurons transduced with LAMP1-3xKBS-NG.

To confirm that the reduction of *RPS7* and *RPS27A* mRNAs in the axon of BORCS5-KO i3Neurons was due to the inability of lysosome-related vesicles to access the axon, we examined the effect of driving movement of lysosome-related vesicles in these neurons by expressing LAMP1 fused with three copies of a kinesin-binding sequence (KBS) from PLEKHM2 (LAMP1–3×KBS). This manipulation enabled direct coupling of lysosomes to kinesin-1 in the absence of BORC^31,47^. We observed that expression of LAMP1–3×KBS restored the presence of both *RPS7* and *RPS27A* mRNAs within the axon of BORCS5-KO i3Neurons, most noticeably in the central domain of axonal growth cones (Extended Data Figs. 6a,b and 7a,b).

### BORC-KO decreases translation of mRNAs in the axon

**Fig. 5:**
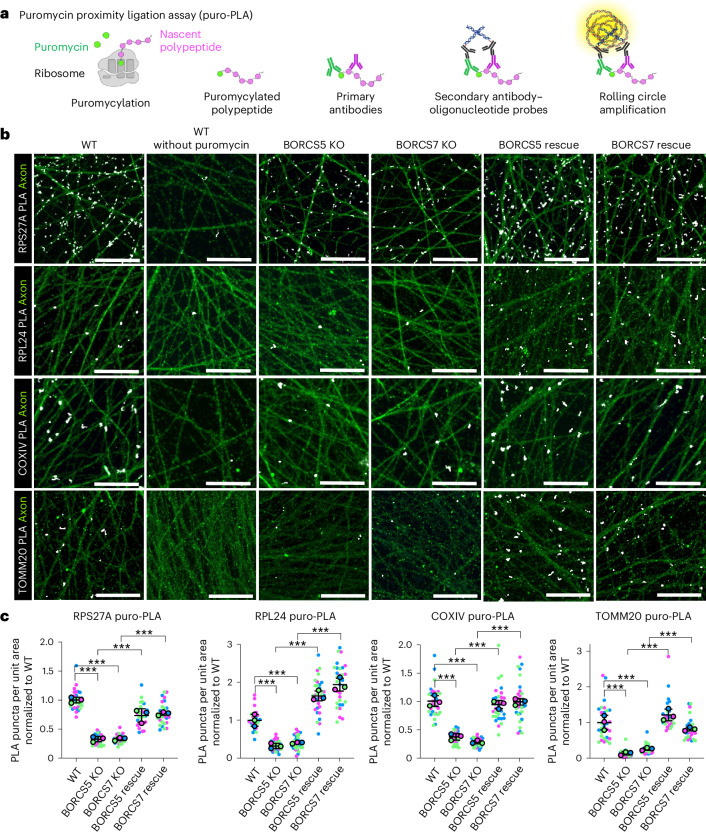
Decreased translation of mRNAs encoding ribosomal and mitochondrial proteins in axons from BORC-KO neurons. **a**, Schematic representation of puro-PLA (see Methods for description)^48^. **b**, WT, BORCS5-KO, BORCS7-KO, BORCS5-KO rescue and BORCS7-KO rescue i3Neurons grown for 25 d on glass coverslips were double stained with puro-PLA for the ribosomal proteins RPS27A or RPL24, or the mitochondrial proteins COXIV or TOMM20 (all in grayscale), and MAP2 for faint axonal staining as described above (green channel). The axonal field was imaged by confocal fluorescence microscopy. Scale bars, 20 μm. The experiment was repeated three times. **c**, Quantification of the number of puro-PLA puncta per unit axon area from three independent experiments such as that shown in **b**. Results are represented as SuperPlots^77^ showing the individual data points in different colors, the mean from each experiment and the mean of the means ± s.d. Statistical significance was calculated by one-way ANOVA with Tukey’s multiple comparisons test. RPS27A: BORCS5 KO versus WT ***P < 0.001, BORCS7 KO versus WT ***P < 0.001, BORCS5 rescue versus BORCS5 KO ***P < 0.001, BORCS7 rescue versus BORCS7 KO ***P < 0.001. RPL24: BORCS5 KO versus WT ***P < 0.001, BORCS7 KO versus WT ***P < 0.001, BORCS5 rescue versus BORCS5 KO ***P < 0.001, BORCS7 rescue versus BORCS7 KO ***P < 0.001. COXIV: BORCS5 KO versus WT ***P < 0.001, BORCS7 KO versus WT ***P < 0.001, BORCS5 rescue versus BORCS5 KO ***P < 0.001, BORCS7 rescue versus BORCS7 KO ***P < 0.001. TOMM20: BORCS5 KO versus WT ***P < 0.001, BORCS7 KO versus WT ***P < 0.001, BORCS5 rescue versus BORCS5 KO ***P < 0.001, BORCS7 rescue versus BORCS7 KO ***P < 0.001.

To examine the impact of the depletion of mRNAs on the synthesis of ribosomal and mitochondrial proteins in BORC-KO axons, we performed puromycin labeling coupled with a proximity ligation assay (puro-PLA) (Fig. 5a)^48^. These experiments revealed translation of mRNAs encoding the ribosomal subunits RPS27A and RPL24, and the mitochondrial proteins COXIV and TOMM20, as puncta in the axons of WT i3Neurons (Fig. 5b). Because the mRNAs encoding these proteins were reduced in BORCS5-KO axons relative to WT axons (Supplementary Tables 1 and 2), we expected a reduction of their synthesis in BORC-KO axons. Indeed, we observed that the number of puro-PLA puncta for all four proteins was reduced in the axons of BORCS5-KO and BORCS7-KO i3Neurons and recovered upon re-expression of the corresponding BORC subunits (Fig. 5b,c). These results demonstrated a reduction in the axonal synthesis of ribosomal and mitochondrial proteins whose mRNAs were decreased upon BORC KO.

### Altered properties of axonal mitochondria in BORC-KO i3Neurons

**Fig. 6:**
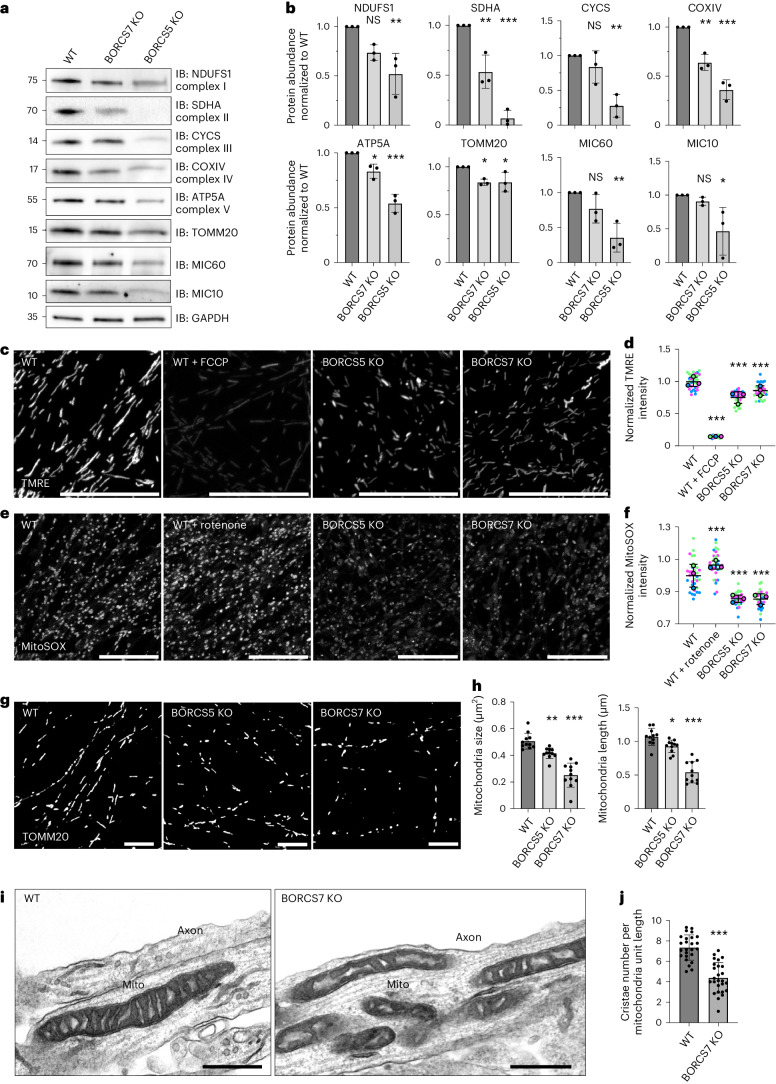
Mitochondrial defects in axons from BORC-KO i3Neurons. **a**, IB analysis of axons from WT, BORCS5-KO or BORCS7-KO i3Neurons cultured in microfluidic devices and analyzed for endogenous mitochondrial proteins and GAPDH (loading control). Molecular mass markers (in kDa) are indicated on the left. **b**, Quantification from three independent experiments such as that shown in **a**. Statistical significance was calculated by one-way ANOVA with Dunnett’s multiple comparisons test. Data are represented as mean ± s.d. *P* values relative to WT: NDUFS1, BORCS7 KO *P* = 0.078, BORCS5 KO **P = 0.006. SDHA, BORCS7 KO **P = 0.003, BORCS5 KO ***P < 0.001. CYCS, BORCS7 KO *P* = 0.417, BORCS5 KO **P = 0.003. COXIV, BORCS7 KO **P = 0.002, BORCS5 KO ***P < 0.001. ATP5A, BORCS7 KO *P = 0.026, BORCS5 KO ***P < 0.001. TOMM20, BORCS7 KO *P = 0.031, BORCS5 KO *P = 0.033. MIC60, BORCS7 KO *P* = 0.231, BORCS5 KO **P = 0.005. MIC10, BORCS7 KO *P* = 0.811, BORCS5 KO *P = 0.034. **c**, WT i3Neurons were incubated with the mitochondrial ΔΨm-reporter TMRE with or without FCCP (control), and the axonal field was imaged live. Scale bars, 20 μm. **d**, Quantification of TMRE intensity per unit axonal area from *n* = 3 independent experiments such as that shown in **c**. Data are represented as SuperPlots^77^ showing the individual data points, the mean from each experiment and the mean of the means ± s.d. Statistical significance was calculated by one-way ANOVA with Dunnett’s multiple comparisons test. *P* values relative to WT: TMRE, WT + FCCP ***P < 0.001, BORCS5 KO ***P < 0.001, BORCS7 KO ***P < 0.001. **e**, i3Neurons were incubated with MitoSOX with or without rotenone (control), and the axonal field was imaged live. Scale bars, 20 μm. **f**, Quantification of MitoSOX intensity per unit area of axonal field as described for **d**. Statistical significance was calculated by one-way ANOVA with Dunnett’s multiple comparisons test. *P* values relative to WT: MitoSOX, WT + rotenone ***P < 0.001, BORCS5 KO ***P < 0.001, BORCS7 KO ***P < 0.001. **g**, i3Neurons were immunostained for TOMM20, and the axonal field was imaged. Scale bars, 10 μm. **h**, Size and length of axonal mitochondria measured from experiments such as that shown in **g**. Values are the mean ± s.d. from ~12 fields. Statistical significance was calculated by one-way ANOVA with Dunnett’s multiple comparisons test. *P* values relative to WT: Size, BORCS5 KO **P < 0.006, BORCS7 KO ***P < 0.001. Length, BORCS5 KO *P < 0.025, BORCS7 KO ***P < 0.001. **i**, Axonal mitochondria were analyzed by TEM in *n* = 2 independent experiments. Scale bars, 400 nm. **j**, Quantification of the number of cristae per mitochondrial unit length from *n* = 26 axons in *n* = 2 independent experiments. Values are the mean ± s.d. from images like the ones in **i**. Statistical significance was calculated using unpaired two-tailed Student’s *t*-test. BORCS7 KO versus WT ***P < 0.001. Source data

Next, we examined the impact of decreased mRNA levels on the levels of ribosomal and mitochondrial proteins in the axon. We were unable to detect ribosomal proteins by immunoblot analysis of axonal fractions. However, we could readily detect various mitochondrial proteins in the same fractions. We observed that the levels of the mitochondrial complex I–V proteins NDUFS1, SDHA, CYCS, COXIV and ATP5A were significantly reduced in axons from BORCS5-KO and BORCS7-KO i3Neurons, with only two exceptions in which the reductions did not reach statistical significance (NDUFS1 and CYCS in BORCS7-KO cells) (Fig. 6a,b). Axons from BORCS5-KO i3Neurons also exhibited reduced levels of the MIC60 and MIC10 components of the mitochondrial contact site and cristae organizing system (MICOS) in the inner mitochondrial membrane (Fig. 6a,b). In axons from BORCS7-KO i3Neurons, the reductions in MICOS proteins did not reach statistical significance (Fig. 6a,b). Thus, there was an overall trend toward lower levels of mitochondrial proteins in the BORC-KO axons, although these reductions were less dramatic than those of the corresponding mRNAs (Supplementary Tables 1 and 2).

Because complexes I, III and IV of the OXPHOS system are proton pumps that generate the mitochondrial membrane potential (ΔΨm), we hypothesized that the lower levels of components of these complexes could decrease ΔΨm in axons from BORC-KO i3Neurons. Indeed, we observed that, although mitochondria were still present in axons from BORCS-KO i3Neurons (Fig. 1d), they displayed significantly reduced staining for the ΔΨm reporter tetramethylrhodamine ethyl ester (TMRE) (Fig. 6c,d). Because some studies showed a positive correlation between ΔΨm and production of ROS^49^, we also examined ROS levels in the axon of WT and KO i3Neurons by staining with MitoSOX (Fig. 6e,f). We observed that axonal mitochondria from BORCS5-KO and BORCS7-KO i3Neurons also exhibited reduced MitoSOX fluorescence (Fig. 6e,f).

Although mitochondria were present in the axons of BORC-KO i3Neurons, morphological analyses of immunofluorescence microscopy images showed that, on average, they had reduced surface area and length (Fig. 6g,h). In addition, transmission electron microscopy (TEM) of i3Neurons showed that, whereas WT axonal mitochondria displayed a regular spacing of their cristae, BORCS7-KO axonal mitochondria exhibited misaligned and deformed cristae (Fig. 6i). The number of cristae was also reduced in BORCS7-KO i3Neurons (Fig. 6j).

Taken together, these experiments demonstrated that axonal mitochondria are defective in BORC-KO i3Neurons, a phenotype that is likely secondary to the depletion of lysosome-related vesicles transporting mRNAs for synthesis of mitochondrial proteins.

### Accumulation of autophagosomes and swellings in BORC-KO axons

**Fig. 7:**
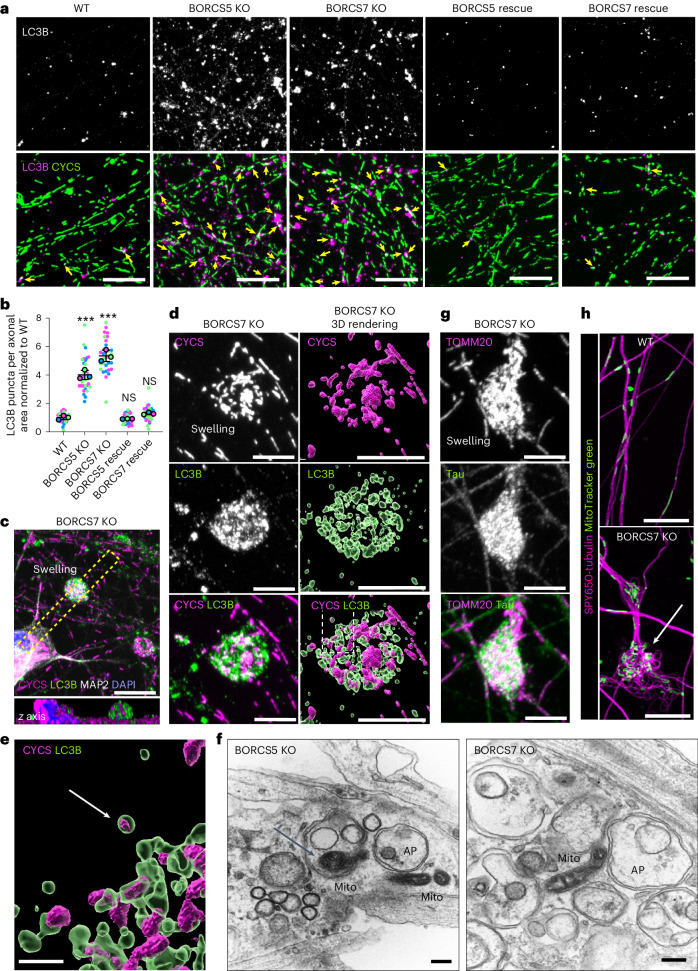
Accumulation of axonal autophagosomes and swellings in BORC-KO i3Neurons. **a**, i3Neurons were immunostained for LC3B (autophagosomes) and CYCS (mitochondria), and the axonal fields were imaged. Single-channel images are shown in grayscale. Arrows in the merge panels indicate LC3B puncta in association with mitochondria. Scale bars, 10 μm. **b**, Quantification of the number of LC3B puncta per unit of axonal area from three independent experiments, such as those shown in **a**. Data are represented as SuperPlots^77^ showing the individual data points, the mean from each experiment and the mean of the means ± s.d. Statistical significance was calculated by one-way ANOVA with Dunnett’s multiple comparisons test. Significance relative to WT: BORCS5 KO ***P < 0.001, BORCS7 KO ***P < 0.001, BORCS5 KO rescue *P* < 0.981, BORCS7 KO rescue *P* < 0.468. **c**, BORCS7-KO i3Neurons were quadruple stained for LC3B, CYCS, MAP2 and nuclear DNA (DAPI). The top image shows axonal swellings in *x*–*y* view. Scale bar, 10 μm. The bottom image shows a *z* axis view of the dashed rectangle. **d**, 3D volume rendering using Imaris of a swelling from a BORCS7-KO i3Neuron immunostained for LC3B and CYCS as above. Scale bars, 5 μm. **e**, Zoomed-in view of the boxed area in the 3D rendering from **d** shows a mitochondrion inside an autophagosome (white arrow). Scale bar, 1 μm. **f**, TEM of axons showing swellings filled with autophagosomes (AP) and mitochondria (Mito). Arrow indicates a mitochondrion inside an autophagosome. Scale bars, 200 nm. **g**, Swelling from a BORCS7-KO i3Neuron immunostained for TOMM20 and the microtubule-associated protein Tau. Scale bars, 5 μm. See also Extended Data Fig. 8a. **h**, i3Neurons sequentially stained with SPY650-tubulin and MitoTracker Green (mitochondria) were imaged live on an Airyscan confocal microscope. Arrow points at microtubule swirls in an axonal swelling. Scale bars, 10 μm. See also Extended Data Fig. 8b. All neurons shown in **c**–**h** were grown for 25 d, and the experiments were repeated three times.

**Extended Data Fig. 8:**
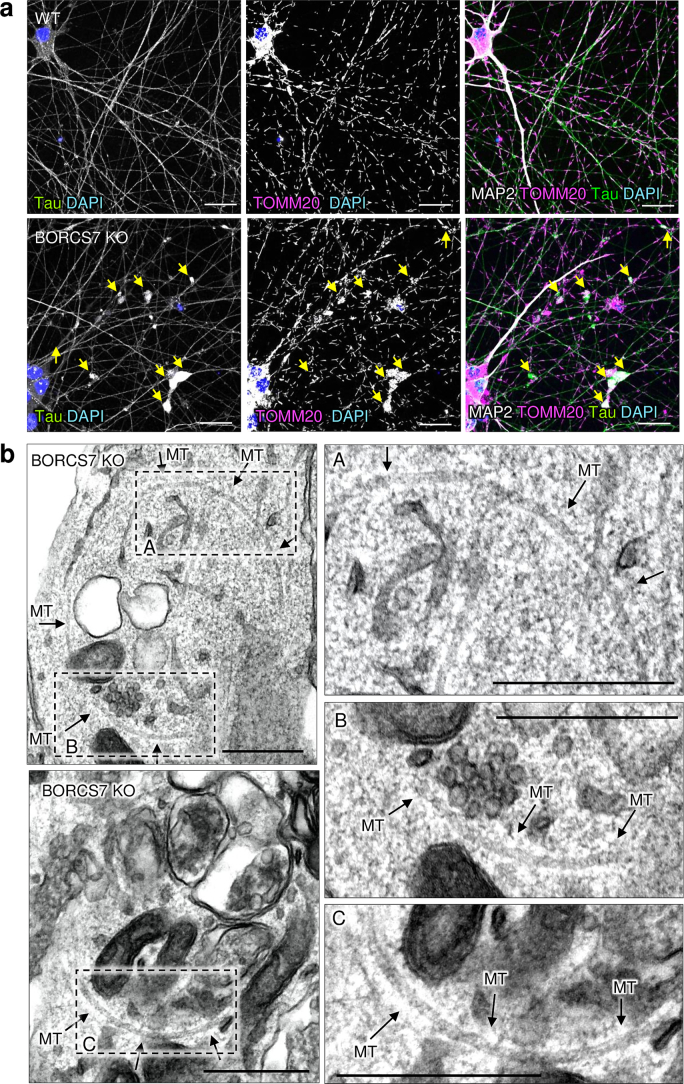
Tau aggregates and microtubule swirls in axonal swellings from BORCS7-KO i3Neurons. **a**, WT and BORCS7-KO i3Neurons were cultured for 25 days on glass coverslips and stained with Tau-1 antibody (marker for axons and axonal degeneration when aggregated), TOMM20 (mitochondria), and MAP2 (soma and dendrites). Nuclei were stained with DAPI (blue). Neurons were imaged by confocal fluorescence microscopy. Notice that swellings in BORCS7-KO axons (yellow arrows) contain Tau-1-positive aggregates and mitochondria. Scale bars: 10 μm. Experiments were repeated three times. **b**, BORCS7-KO i3Neurons grown for 25 days on glass coverslips were analyzed by TEM. *Left column*: detailed view of two axonal swellings in BORCS7-KO axons. *Right column*: magnified views of the boxed areas on the left. Curved microtubules (MT) indicative of a swirl-like organization are visible in the swellings (arrows). Scale bars: 400 nm. This experiment was repeated twice.

Loss of mitochondrial ΔΨm is known to trigger mitophagy^50^. Immunofluorescence microscopy analyses indeed showed that BORCS5 or BORCS7 KO caused a marked increase in axonal puncta stained for the autophagosome marker LC3B^51^ (Fig. 7a,b), as previously observed in other models of axonal dystrophy and degeneration^52–54^. The LC3B increase was reversed by re-expression of BORCS5 or BORCS7 in the corresponding BORC-KO i3Neurons (Fig. 7a,b). In BORC-KO i3Neurons, the LC3B puncta were often found in association with mitochondria stained for CYCS (Fig. 7a, yellow arrows). LC3B accumulation was most striking in axonal swellings, which were numerous in BORC-KO i3Neurons (Fig. 7c,d and Extended Data Fig. 8a). The swellings contained many small mitochondria (Fig. 7d), which, in some cases, were engulfed within LC3B-positive autophagosomes (Fig. 7e, arrow). TEM also showed accumulation of autophagosomes and mitochondria in axonal swellings of BORC-KO i3Neurons, with some mitochondria found within the autophagosomes (Fig. 7f, arrow). In addition, the axonal swellings were heavily stained for Tau (Fig. 7g) and exhibited an aberrant swirl-like organization of microtubules (Fig. 7h and Extended Data Fig. 8b), both characteristic of axonal degeneration^55,56^.

**Fig. 8:**
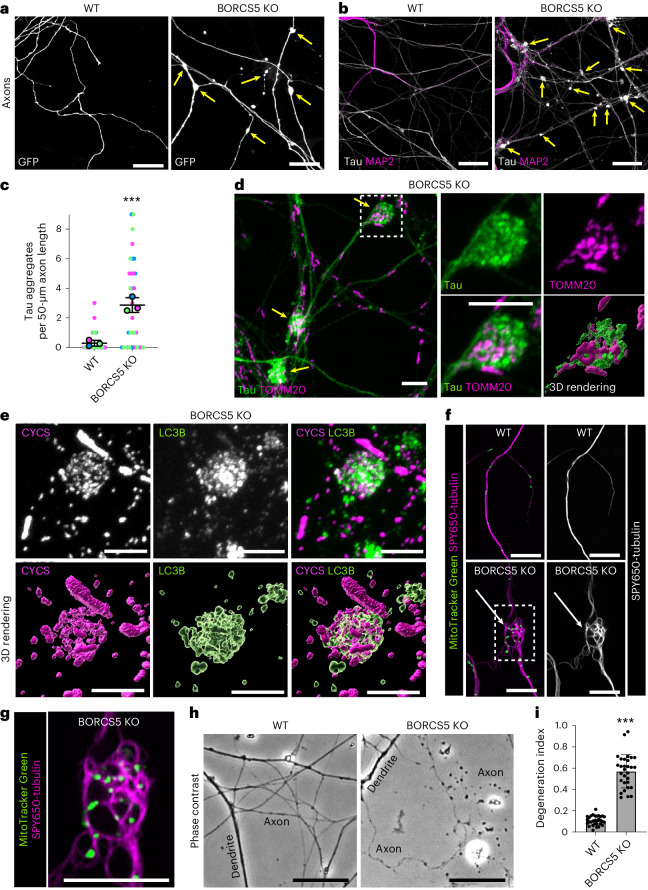
Accumulation of axonal autophagosomes and swellings in cortical neurons from BORCS5-KO mice. **a**, Cortical neurons isolated from WT and BORCS5-KO E17 mouse embryos^11^ were transfected with a plasmid expressing soluble GFP. Arrows indicate axonal swellings. Scale bars, 20 μm. The experiment was repeated three times. **b**, Cortical neurons from WT and BORCS5-KO mice were immunostained for Tau (axon) and MAP2 (soma and dendrites). Arrows indicate the accumulation of Tau-positive aggregates in the swellings. Scale bars, 20 μm. **c**, Quantification of the number of Tau-positive swellings per unit length of axon from three experiments, such as shown in **b**. Data are represented as SuperPlots^77^ showing the individual data points, the mean from each experiment and the mean of the means ± s.d. Statistical significance was calculated using an unpaired two-tailed Student’s *t*-test. BORCS5 KO versus WT ***P < 0.001. **d**, Axonal swellings (arrows) in BORCS5-KO mouse cortical neurons immunostained with antibodies to Tau and TOMM20 (mitochondria). The four images on the right are magnified views and a 3D rendering of the boxed area. Scale bars, 5 μm. **e**, Immunofluorescence microscopy and 3D rendering of axonal swellings in cortical neurons from BORCS5-KO mice stained for LC3B (autophagosomes) and CYCS (mitochondria). Scale bars, 5 μm. **f**, Axons from WT and BORCS5-KO mouse cortical neurons sequentially stained with SPY650-tubulin (tubulin) for 1 h and MitoTracker Green (mitochondria) for 10 min were imaged live on an Airyscan confocal microscope. Arrows point to microtubule swirls in an axonal swelling. Scale bars, 10 μm. **g**, Zoomed-in view of the boxed area from **f** shows microtubule swirls in an axonal swelling. Scale bar, 20 μm. All the experiments shown in **d**–**f** were repeated three times. **h**, Phase-contrast microscopy of axons from WT and BORCS5-KO mouse cortical neurons. Notice fragmentation in the BORCS5-KO axons. Scale bars, 5 μm. **i**, Quantification of axon DI from two independent experiments, such as the one shown in **h**. Values are the mean ± s.d. from 30 images. Statistical significance was calculated using an unpaired two-tailed Student’s *t*-test. BORCS5 KO versus WT ***P < 0.001. Neurons shown in this figure were cultured for 7 d (**a**–**g**) or 10 d (**h** and **i**).

We corroborated some of these findings in day-in-vitro (DIV) 7 cortical neurons from homozygous BORCS5-KO mouse embryonic day (E) 17 embryos^11^. We observed that, like the BORCS5-KO human axons, BORCS5-KO mouse axons displayed swellings (Fig. 8a) containing Tau, mitochondria, LC3B (Fig. 8b–e) and microtubule swirls (Fig. 8f,g). Although axon fragmentation was not apparent in BORCS5-KO i3Neurons for up to 45 d in culture, it was clearly seen in BORCS5-KO mouse neurons cultured for 10 d (Fig. 8h,i). This difference could indicate that native mouse neurons are more vulnerable or represent a different developmental stage than in vitro differentiated i3Neurons.

**Extended Data Fig. 9:**
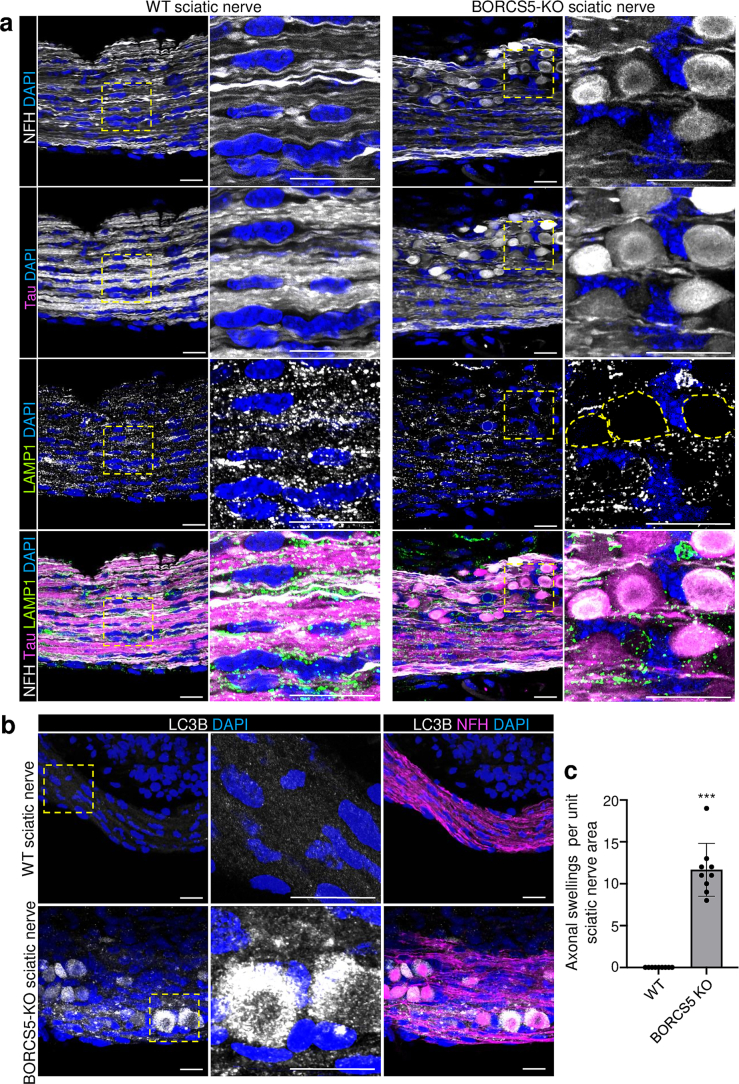
Axonal swellings containing neurofilament protein, Tau, and autophagosomes in sciatic nerves from BORCS5-KO mice. **a**, **b**, Sciatic nerves were isolated from WT and BORCS5-KO E17 mouse embryos. Nerves were fixed and immunostained for endogenous Tau (magenta) and LAMP1 (lysosomes, green) (panel **a**) or LC3B (autophagosomes, grayscale) (panel **b**). Nerves were also co-stained for NFH (axons, grayscale in panel **a** and magenta in panel **b**), and DAPI (nuclei, blue), and imaged by confocal fluorescence microscopy. Enlarged views of the boxed areas are shown on the right. Areas encircled by dashed lines indicate swellings devoid of LAMP1 staining. Scale bars: 20 μm. **c**, Quantification of the number of axonal swellings (NFH-positive) per unit of sciatic nerve area from n = 9 images captured from n = 3 animals. Values are the mean ± SD from representative images like those shown in panels **a** and **b**. Statistical significance was calculated using an unpaired two-tailed Student’s t test. BORCS5 KO vs WT ***P < 0.001.

**Extended Data Fig. 10:**
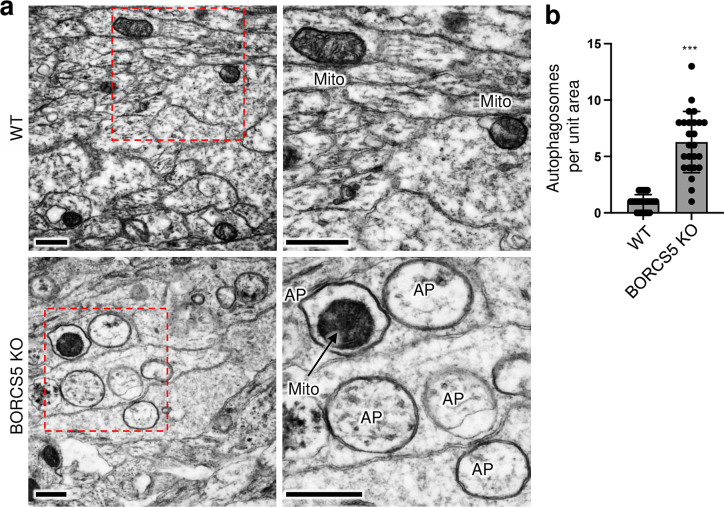
Accumulation of autophagosomes in the axons of BORCS5 -KO mouse brain. **a**, Brains were harvested from WT and BORCS5-KO E17 mouse embryos and the corpus callosum was analyzed by TEM as described in the Methods. The figure shows low magnification (left column) and high magnification images of the boxed areas (right column). Notice the presence of numerous autophagosomes (AP) in the axons of BORCS5-KO neurons, morphologically identified by their size and presence of synaptic vesicles, microtubules, and neurofilaments. Scale bars: 600 nm. **b**, Quantification of the number of autophagosomes per field unit area from n = 25 images captured from n = 2 animals. Values are the mean ± SD from representative images like those shown in panel **a**. Statistical significance was calculated using an unpaired two-tailed Student’s t test. BORCS5 KO vs WT ***P < 0.001.

Finally, immunohistochemical analysis of the sciatic nerve also showed the presence of numerous swellings labeled for the axonal marker NFH, Tau and LC3B but not LAMP1 in BORCS5-KO axons (Extended Data Fig. 9a–c). Furthermore, TEM revealed the presence of autophagosomes, some containing engulfed mitochondria, in axons from the corpus callosum of BORCS5-KO but not WT mouse E17 embryos (Extended Data Fig. 10a,b).

We conclude that BORC KO in both human and mouse neurons causes accumulation of axonal autophagosomes in association with mitochondria, suggestive of increased mitophagy. This accumulation is particularly striking in axonal swellings containing Tau and microtubule swirls, all conditions that are likely associated with axonal dysfunction and degeneration.

## Discussion

Our study demonstrates that preventing transport of lysosome-related vesicles into the axon by BORC KO causes depletion of a large subset of mRNAs from the axon but not the whole neuron. This subset comprises many nuclear-encoded mRNAs encoding ribosomal and mitochondrial proteins, likely as a result of reduced RNA granule association with and hitchhiking on lysosomal vesicles^19,23^. Depletion of these mRNAs is linked to decreased synthesis of ribosomal and mitochondrial proteins as well as alterations in mitochondrial morphology, ΔΨm, ROS and autophagy in the axon. These defects likely underlie the development of axonal swellings as a prelude to axonal degeneration and fragmentation. This series of events probably explains the neurodevelopmental/neurodegenerative phenotype of mice with homozygous mutations^7,11,57^ and human patients with biallelic variants^33–35^ of BORC-subunit genes. Pathway analysis of the depleted mRNAs also suggests similarities in the pathogenetic mechanisms of BORC deficiency and common neurodegenerative diseases.

BORC is a component of an ensemble that mediates coupling of lysosomes to kinesin-1 and kinesin-3 microtubule motors^6,31,32^. Although the properties of axonal anterograde lysosome-related vesicles differ from those of conventional lysosomes^5,9,12–14^, they also depend on BORC for transport into the axon^6,7,9–11,30^. The results presented here and in a previous study^19^ show that one function of these vesicles is to carry RNA granules and their mRNA cargo into the axon. Coupling of RNA granules to microtubule motors has also been shown to be mediated directly by interaction of RNPs with the motors^27–29^ or indirectly via other organelles, such as mitochondria^24,25^, late endosomes/lysosomes^19,23^ or early endosomes^23,26^. Each of these mechanisms may account for the axonal transport of different mRNA subsets. The specific depletion of axonal lysosome-related vesicles in BORC-KO i3Neurons allowed us to define the mRNA subset dependent on these vesicles for transport into the axon. This subset is large and comprises mRNAs that are normally abundant in the axon, such as those encoding ribosomal and mitochondrial proteins.

Although most ribosome assembly occurs in the nucleolus, some ribosomal proteins may be added or exchanged immediately after export from the nucleus into the cytoplasm^58–60^. In neurons, some ribosomes are transported into the axon, to some extent also by hitchhiking on lysosomal or late endosomal vesicles^23^. The deployment of ribosomes to the axon enables local synthesis of proteins important for axon maintenance and synaptic function^61–65^. Over time, ribosomal proteins may become damaged^66^. Rather than targeting the whole ribosome for destruction, local translation in the axon may allow for the replacement of the damaged proteins^58,67,68^. Moreover, new synthesis of ribosomal proteins in the axon may enable remodeling of ribosomes in response to physiological or pathological conditions.

The subset of mRNAs depleted in BORC-KO axons also comprises many mRNAs encoding mitochondrial proteins. These include nuclear-encoded and mitochondria-encoded subunits of all five complexes of the electron transport chain as well as nuclear-encoded MICOS components, the mitochondrial import system and mitophagy. As discussed above for ribosomes, these mRNAs are likely translated locally for repair of mitochondria, which are particularly prone to oxidative damage or proteotoxic stress^62,69^. Probably because of reduced synthesis of the corresponding proteins, axonal mitochondria are smaller, have reduced ΔΨm, lower ROS and disorganized cristae and exhibit increased association with autophagosomes. These are all characteristics of dysfunctional mitochondria that are targeted for degradation by mitophagy^70^. However, mitophagy is likely arrested at the stage of autophagosomes because of the absence of axonal lysosomes to confer degradative activity and/or retrograde motility upon fusion^6,10,15^. The accumulation of abnormal mitochondria and autophagosomes is most dramatic in axonal swellings, which may correspond to the eosinophilic spheroids previously observed in nerves from BORCS5-KO mouse embryos^11^. The swellings in BORC-KO human i3Neurons, mouse cortical neurons and sciatic nerves also exhibit accumulation of Tau and microtubule swirls characteristic of other neurodegenerative conditions^55,56^. Indeed, axons from BORCS5-KO mouse cortical neurons undergo fragmentation after 10 d in culture, representing the final stage of a degenerative cascade likely caused by the depletion of axonal lysosome-related vesicles.

In addition to mRNAs encoding ribosomal and mitochondrial proteins, BORC-KO causes depletion of mRNAs encoding cytoskeletal proteins, proteasome subunits, ferritin heavy chain and RBPs from the axon. Moreover, BORC-KO increases the axonal abundance of another subset of mRNAs mainly encoding nuclear, lysosomal, endoplasmic reticulum and cell adhesion proteins. The increased mRNAs could be transported into the axon by coupling of RNA granules to microtubule motors or other organelles. Moreover, BORC normally promotes the recruitment of other ARL8 effectors, including the dynein–dynactin adaptor protein RUFY3 (refs. ^71,72^) and the endolysosomal tethering complex HOPS^73^, to lysosomes. Alterations in all these processes, along with the loss of other lysosomal functions such as degradation or signaling, could additionally contribute to mitochondrial dysfunction and axon degeneration in BORC-KO neurons.

Biallelic variants in BORC subunits have been shown to cause neurodevelopmental or early-infantile neurodegenerative disorders^33–35^. Our results suggest that CNS defects in BORC-variant patients may involve a pathogenetic mechanism like that described in this study. KEGG analysis further suggests a connection between the pathogenetic mechanism of BORC deficiency and those of adult neurodegenerative disorders. What these diseases have in common is the presence of mitochondrial abnormalities, specifically related to OXPHOS, mitophagy and fusion–fission^74–76^. BORC deficiency may, thus, trigger common neurodegenerative pathways at an early age.

In summary, our findings demonstrate that axonal anterograde lysosome-related vesicles carry a large subset of mRNAs, predominantly encoding ribosomal and mitochondrial proteins. It is likely that local translation of these mRNAs enables the replacement of damaged ribosomal and mitochondrial proteins, ultimately contributing to the maintenance of ribosomal and mitochondrial homeostasis in the axon.

## Methods

### Reagent sources

Sequences of oligonucleotides, company names and catalog numbers for reagents used in this paper are listed in Supplementary Table 3.

### Human iPSC culture and neuronal differentiation

Human iPSCs expressing doxycycline-inducible *NGN2* (neurogenin)^36,37^ were cultured on Matrigel-coated dishes in Essential 8 Flex Medium (E8Flex) (Thermo Fisher Scientific) according to the manufacturer’s instructions. In brief, cells were passaged with Accutase (STEMCELL Technologies) when 70% confluent and seeded into E8Flex supplemented with 10 μM Y-27632 dihydrochloride ROCK inhibitor (S1049, Selleck Chemicals). After 24 h, the medium was replaced with E8Flex without ROCK inhibitor and maintained until the next passage. Differentiation of iPSCs to i3Neurons was performed as described^36,37^. iPSCs were seeded on day 0 in induction medium containing DMEM/F12 (11330032, Gibco, Thermo Fisher Scientific) with 1× non-essential amino acids (NEAAs) (11140050, Gibco, Thermo Fisher Scientific), 1× GlutaMAX (35050061, Gibco, Thermo Fisher Scientific), 1× N2A supplement (17502048, Gibco, Thermo Fisher Scientific), 2 μg ml^−1^ doxycycline (D9891, MilliporeSigma) and 10 μM Y-27632 dihydrochloride ROCK inhibitor (S1049, Selleck Chemicals). The induction medium was changed every day for 3 d. Cells were then lifted with Accutase, counted and plated in neuronal culture medium consisting of BrainPhys medium (5790, STEMCELL Technologies) supplemented with 10 ng ml^−1^ neurotrophin-3 (450-03, PeproTech, Thermo Fisher Scientific), 10 ng ml^−1^ BDNF (450-02, PeproTech, Thermo Fisher Scientific), 1× B-27 supplement serum free (17504044, Gibco, Thermo Fisher Scientific), 2 μg ml^−1^ doxycycline (D9891, MilliporeSigma) and 1 μg ml^−1^ mouse laminin (23017015, Gibco, Thermo Fisher Scientific). The coverslips for growth of i3Neurons were pre-treated with poly-l-lysine hydrobromide (P2636, MilliporeSigma) and laminin (11243217001, Roche, MilliporeSigma). Every 3 d, half of the medium was removed, and an equal volume of fresh medium was added to maintain neuronal health.

### Mouse husbandry

All mouse procedures were conducted following the National Institutes of Health Guide for the Care and Use of Laboratory Animals, under protocol 21-021 that received ethical approval by the Eunice Kennedy Shriver National Institute of Child Health and Human Development (NICHD) Animal Care and Use Committee. Mouse husbandry is described in detail in Supplementary Data 1.

### Culture of primary cortical neurons

Primary cortical neurons were prepared from mice as previously described^78^. In brief, after timed pregnancies, E17 mice were harvested and euthanized by decapitation. The brain was collected, and meninges were removed, after which cortices were isolated in sterile HBSS (20 mM HEPES, pH 7.5). Cortices were then treated with 0.25% trypsin (Gibco, Thermo Fisher Scientific) and 100 μg ml^−1^ DNase (Roche) for 15 min at 37 °C. To stop trypsin enzymatic action, one volume of DMEM without phenol red (Thermo Fisher Scientific), supplemented with 4.5 g L^−1^ glucose, 25 mM HEPES, 10% heat-inactivated horse serum (Gibco, Thermo Fisher Scientific) and 100 U ml^−1^ penicillin–streptomycin (Quality Biological) (adhesion medium), was added. The tissue was then pipetted through a 10-ml serological pipette to disrupt it mechanically. Single cells were then strained through a 70-μm nylon filter (Corning) and centrifuged at 700*g* for 10 min. The cell pellet was resuspended in 5 ml of adhesion medium, and cells were counted. Cells (80,000) were plated on 18-mm glass coverslips previously coated with polylysine and laminin. After 3 h, the medium was changed to complete Neurobasal medium (CNB) consisting of Neurobasal medium (Gibco, Thermo Fisher Scientific) supplemented with 1× B27 serum free (Thermo Fisher Scientific), 4.5 g L^−1^ glucose (Quality Biological) and 100 U ml^−1^ penicillin–streptomycin (Quality Biological). For timed pregnancies, mice of both sexes of approximately 6 weeks of age were used.

### CRISPR–Cas9 KO of *BORCS5* or *BORCS7*

To inactivate the *BORCS5* and *BORCS7* genes in iPSCs, we used the CRISPR–Cas9 system with a single guide RNA (sgRNA) per gene. For *BORCS5* KO, the sgRNA sequence was designed using Benchling (https://benchling.com) to target the second exon. We designed one forward and one reverse oligonucleotide bearing the selected sgRNA (uppercase) and the sequence necessary for cloning into the px458 plasmid (lowercase): forward 5′-caccgCTCAGGGCTCCCAGGCCTCA and reverse 5′-aaacTGAGGCCTGGGAGCCCTGAGc. For *BORCS7* KO, we used Benchling to design oligos bearing an sgRNA sequence targeting exon 1: forward 5′-caccgACGGAGAAGGTGACCACCTG and reverse 5′-aaacCAGGTGGTCACCTTCTCCGTc. Each of the guides was separately cloned into px458 plasmid (gift from F. Zhang, Addgene plasmid 48138). In brief, px458 plasmid was cut with BbsI, and the annealed oligonucleotides were inserted using T4 DNA ligase. The insertion was verified by sequencing. The plasmids bearing the sgRNAs were co-transfected into iPSCs using Lipofectamine 2000 (Thermo Fisher Scientific). Forty-eight hours later, single cells were sorted into 96-well plates based on GFP-positive signal. The single cells were allowed to grow in complete E8Flex medium supplemented with RevitaCell (A2644501, Thermo Fisher Scientific) for 1–2 weeks. When colonies were visible, cells were transferred to a six-well dish and grown until confluency. Cells were then harvested, and genomic DNA was isolated. PCR was conducted using forward 5′-AGTGACTCCTTCACCAGCCAAGCAT and reverse 5′-gtagagggaaagTAGTAGGGCTACAC primers for *BORCS5* and forward 5′-GGCCCCGGCGACTCACCATCGTCAG and reverse 5′-TACAATTCCCAAGATGCAACGCGAC primers for *BORCS7*. The PCR products were sequenced to verify the change from the WT sequence. Further verification was conducted by SDS-PAGE and immunoblotting with antibodies to BORCS5 or BORCS7 (Fig. 1c).

### Lentivirus generation and rescue of KO cells

To rescue BORCS5-KO and BORCS7-KO cells, we used a lentiviral PGK-eGFP plasmid in which the human PGK promoter was substituted by the human EF1α promoter. The human BORCS5 or BORCS7-HA sequence was inserted after the promoter. A self-cleaving 2a peptide (P2A) was added between the BORC protein and the C-terminal eGFP. All these changes were carried out using Gibson Assembly (New England Biolabs). We also used site-directed mutagenesis to change glycine-2 to alanine in BORCS5, a mutation that was previously shown to prevent N-terminal myristoylation and association of BORCS5 with lysosomes^31^. Lentivirus was produced by co-expressing the obtained plasmid with psPAX2 (Addgene, 12260, gift of Didier Trono), pMD2.G (Addgene, 12259, gift of Didier Trono) and pAdVAntage Vector (Promega) in HEK293T cells using Lipofectamine 3000 (Thermo Fisher Scientific) according to the manufacturer’s instructions. After 36 h, the virus-containing medium was spun at 700*g* for 5 min to remove cell debris and used to infect iPSCs for 36 h. iPSCs were then bulk sorted for GFP signal into a six-well plate. The GFP-positive cells were analyzed by SDS-PAGE and immunoblotting for BORCS5 or BORCS7 to verify the re-introduction of the corresponding protein (Fig. 1c).

### Immunofluorescence microscopy

i3Neurons or primary mouse cortical neurons were seeded on polylysine–laminin-coated 18-mm coverslips or microfluidic devices. Neurons were then fixed in 4% w/v paraformaldehyde (Electron Microscopy Sciences) in PBS for 20 min and permeabilized-blocked with 0.1% w/v saponin and 1% w/v BSA (Gold Bio) in PBS for 20 min. Cells were sequentially incubated with primary and secondary antibodies diluted in 0.1% w/v saponin, 1% w/v BSA in PBS for 30 min at 37 °C (coverslips) or overnight at 4 °C with gentle oscillation (microfluidic devices). Cells were washed three times in PBS. Coverslips were mounted on glass slides using Fluoromount-G (Electron Microscopy Sciences) with DAPI. For the devices, the Fluoromount-G–DAPI was added directly to the cells. *z*-stack cell images were acquired on a Zeiss LSM 880 inverted confocal microscope (Carl Zeiss) using a Plan-Apochromat ×63 objective (numerical aperture (NA) = 1.4). Maximum intensity projections were generated with Zeiss ZEN Black software, and final composite images were created using ImageJ/Fiji (https://fiji.sc/).

### SDS-PAGE and immunoblotting

SDS-PAGE and immunoblotting methods are described in detail in Supplementary Data 1.

### Microfluidic devices

The fabrication of microfluidic devices is described in detail in Supplementary Data 1.

### Plating and culturing of i3Neurons in the microfluidic devices

The i3Neurons pre-differentiated for 3 d as described above were lifted with Accutase and counted. Cells (500,000) were plated in both lateral reservoirs (‘neuron’ compartments; Fig. 2a) of a microfluidic device bonded to a coverslip and pre-coated with polylysine-laminin. Every 3 d, half the medium was removed, and an equal volume of fresh medium was added to maintain neuronal health.

### RNA preparation and sequencing

Samples from the neuronal and axonal compartments of the microfluidic devices were isolated with an RNeasy Micro Kit (Qiagen) according with the manufacturer’s protocol for purification of total RNA from human cells. In brief, RNA from three axonal or neuronal compartments was extracted using a total of 100 μl of the kit’s extraction buffer. No residual axons or cells were observed in the chamber after extraction. No carrier RNA was used for the extraction. On-column DNase digestion was performed. The RNA was eluted from the columns with 10 μl of RNase-free water. Then, 5 μl for axon samples and 1 μl for neuron samples were used for synthesizing cDNAs using a SMART-Seq version 4 Ultra Low Input RNA Kit for Sequencing (Takara). A Nextera XT DNA Library Preparation Kit (Illumina) was used to prepare libraries from the cDNA samples using 1 ng of cDNA. Libraries were run on a NovaSeq (SP Kit version 1.5, 200 cycles). Approximately 1 billion 2×100-bp reads were obtained with sequencing.

### Bioinformatic analyses

Sequenced reads were aligned to GRCh38 human reference GENCODE release 28 using STAR (one-pass) version 2.7.8a^79^. Aligned reads were quantitated using the featureCounts function of the subread package version 2.0.1 (ref. ^80^) with GRCh38 human reference GENCODE release 28. After removing outliers, two WT axons, two WT neurons, three BORCS5-KO axons and three BORCS5-KO neurons were used as biological replicates for downstream differential expression analysis in R version 4.0.3. Differential expression analysis was performed using DESeq2 version 1.30.1 (ref. ^81^) by setting the model to ‘~celltype + 0’ for axon versus neuron comparison in identical genotypes or ‘~genotype + 0’ in identical cell types (for example, axon and neuron). The interaction term was modeled by ‘~celltype + genotype + celltype:genotype + 0’ to compute differentially expressed genes (DEGs) in axons compared to neurons specifically to BORCS5 KO. log_2_ fold changes were computed using ‘normal’ and ‘ashr’ shrinkage in contrasts with and without an interaction term, respectively. DEGs were determined using a false discovery rate (FDR) cutoff of less than 10%.

The number of genes detected per sample was calculated using raw read counts, considering any genes with non-zero read counts in any replicate to be detected. The proportion of protein-coding, ribosomal, mitochondrial ribosomal and other RNA was assessed by matching gene biotypes and gene IDs using the ensembldb version 2.14.0 (ref. ^82^) *Homo sapiens* release ID AH89180 with normalized read counts. MA plots for protein-coding genes were created by plotting mean normalized read counts across the biological replicates on *x* axis and shrunken log_2_ fold changes on *y* axis.

To identify enriched gene sets, functional enrichment analysis on DEGs (FDR < 0.1) was conducted using the functions enrichGO and enrichKEGG from clusterProfiler version 4.8.3 (ref. ^83^). Enrichment dot plots and map were created using the shake_enrichResult, gs_summary_overview and distill_enrichment functions from GeneTonic version 1.5.2 (ref. ^84^). The dot plots were designed to display −log_10_ adjusted *P* values that were computed by enrichGO or enrichKEGG on *x* axis and z-scored by color. The z-scores were calculated by subtracting the number of down genes (log_2_ fold change < 0) from the number of up genes (log_2_ fold change > 0) and dividing the result by the square root of the total number of genes that have changed expression (either up or down). Enriched gene sets in contrasts with an interaction term were computed by performing functional enrichment analysis on DEGs from the interaction contrast that were also not differentially expressed in BORCS5-KO neurons compared to WT neurons.

### Visualization of RNA particle and lysosome movement in the axon

WT and KO iPSCs were co-transfected with a plasmid encoding the PP7 coat protein followed by three HaloTags separated by GB1 domains and an in-house Super PiggyBac Transposase (10.17504/protocols.io.q26g744b1gwz/v1) to ensure stable genomic integration of the HaloTag PP7 coat construct. The cells were allowed to recover in E8Flex medium for at least 1 d before they were passaged and expanded for 3 d. Expanded cells were lifted with Accutase, and, after replating, they were co-transfected again with a plasmid encoding SunTag-*RPS7* or *RPS27A* tagged with 24 PP7 repeats after their 3′ untranslated region (UTR) and the Super PiggyBac Transposase. The polyclonal, stable iPSCs were expanded, differentiated into i3Neurons and plated on 18-mm coverglasses pre-coated with polylysine and laminin as described above. After 15 d in culture, cells were transduced for 36 h with a lentivirus expressing the human LAMP1 protein fused with the monomeric NeonGreen fluorescent protein (LAMP1-mNeonGreen) under the control of the human PGK promoter. In some experiments, cells were similarly transduced with a lentivirus encoding human LAMP1 appended with three repeats of the kinesin-binding sequence (KBS) TNLEWDDSAI from PLEKHM2 (LAMP1–3×KBS)^31,47^, followed by monomeric NeonGreen fluorescent protein (LAMP1–3×KBS-mNeonGreen). Alternatively, the polyclonal, stable iPSCs were expanded and differentiated into i3Neurons as spheroids. For spheroid differentiation, 10,000 iPSCs were lifted with Accutase and resuspended in 20 μl of differentiation medium per sphere. The iPSC suspension (20 μl) was added to a well of an ultra-low-attachment round-bottom 384-well plate (Corning) coated with anti-adherence solution (Life Technologies) for 1 h and washed with PBS three times. Cells were left to sit for 5 min before centrifuging at 540*g* for 2 min to help cells aggregate. The next day, 60 μl of induction medium was added. The third day after addition of induction medium to the 384-well plated cells, spheres were lifted by pipetting with a wide-bore, low-attachment pipette tip. Cells were then re-plated on a four-chamber glass-bottom slide (ibidi) coated with polylysine, followed by a 2-h coating with 15 μg ml^−1^ laminin and supplemented with 250 μl of neuronal culture medium. The day after re-plating, a full medium change was performed, followed by a half medium change 3 d after replating. The spheroids were imaged 1 week after re-plating. For live imaging of i3Neurons in coverslips, cells were incubated overnight with 200 pM of the Halo substrate JF646 (Promega, GA1120), and the axons were imaged live for 60 s (200-ms exposure time, no delay between images) on a spinning disk confocal microscope using a humidified environmental chamber. Alternatively, axons spreading outwards from the spheroids were imaged for 60 s (200-ms exposure time, no delay between images). Imaging of spheroids was focused on an area with clear growth cones and relatively sparse axons to reduce the number of axons crossing over one another.

### puro-PLA

The puro-PLA assay is described in detail in Supplementary Data 1.

### Detection of mRNA using RNAscope ISH

The detection of mRNA using RNAscope ISH assay is described in detail in Supplementary Data 1.

### Measurement of mitochondrial membrane potential and ROS

WT, BORCS5-KO and BORCS7-KO i3Neurons were grown on coverslips for 25 d. To detect the mitochondrial membrane potential (ΔΨm), i3Neurons were stained for 10 min at 37 °C with 50 nM of the ΔΨm-sensitive, fluorescent dye TMRE^85^. As a positive control, we pre-incubated WT i3Neurons for 5 min with 5 μM of the OXPHOS uncoupler carbonyl cyanide-4 (trifluoromethoxy) phenylhydrazone (FCCP) diluted in complete BrainPhys medium. To examine the levels of ROS, we stained i3Neurons for 10 min at 37 °C with 5 μM of the superoxide indicator dye MitoSOX^86^ diluted in complete BrainPhys medium. As a control, we pre-treated WT i3Neurons for 5 min with 1 μM of the mitochondrial complex I inhibitor rotenone. After the incubations, neurons were washed twice with pre-warmed complete BrainPhys medium and imaged live using a Zeiss LSM 780 inverted confocal microscope (Carl Zeiss) fitted with a Plan-Apochromat ×63 objective (NA = 1.4). The microscope was equipped with a humidified environmental chamber kept at 37 °C and 5% CO_2_ to maintain the cells during live imaging.

### Quantification of mitochondria size

The quantification of mitochondrial size is described in detail in Supplementary Data 1.

### Electron microscopy

Electron microscopy methods are described in detail in Supplementary Data 1.

### Immunofluorescence microscopy of mouse sciatic nerves

Sciatic nerves were harvested from E17 WT and BORCS5-KO mouse embryo littermates. Nerves were then fixed for 2 h at 4 °C with 4% paraformaldehyde with gentle agitation. Nerves were permeabilized and blocked overnight with 0.1% w/v saponin and 1% w/v BSA (Gold Bio) in PBS. Nerves were sequentially incubated with primary and secondary antibodies diluted in 0.1% w/v saponin and 1% w/v BSA in PBS overnight at 4 °C with gentle agitation. Nerves were washed three times in PBS for 1 h each and mounted on glass slides using Fluoromount-G–DAPI (Electron Microscopy Sciences). Images were acquired on a Zeiss LSM 880 inverted confocal microscope (Carl Zeiss) using a Plan-Apochromat ×63 objective (NA = 1.4). Final composite images were created using ImageJ/Fiji (https://fiji.sc/).

### Mouse cortical neuron transfection

The transfection of mouse cortical neurons is described in detail in Supplementary Data 1.

### Live imaging of axonal microtubules and mitochondria

WT and KO i3Neurons, and mouse primary cortical neurons, were grown on coverslips for 25 d and 7 d, respectively. Neurons were incubated with SPY650-tubulin (Cytoskeleton, CY-SC503) (1:1,000) in culture medium for 1 h and MitoTracker Green (Thermo Fisher Scientific, M7514) (100 nM) for 10 min at 37 °C. Neurons were washed with pre-warmed culture medium and placed in an environmental chamber at 37 °C and 5% CO_2_ on a Zeiss LSM 880 confocal microscope. Images were taken immediately using Airyscan mode with *z*-stacks and a Plan Apochromat ×63 objective (NA = 1.40).

### Quantification of axonal degeneration

Primary cortical neurons from WT and BORCS5-KO mice were cultured for 10 d on glass coverslips. Phase-contrast light microscopy was used to capture images of axonal regions, identified by their thin diameter and absence of somas. The axon degeneration index (DI) was calculated as described previously^87^. In brief, phase-contrast images were visualized using Fiji, and then the image was transformed into a binary image. Axonal area was marked using the threshold function and measured (total axonal area), followed by particle analysis of the area occupied by fragmented axons. The DI was calculated dividing the area occupied by fragmented axons by the total axonal area.

### Statistics and reproducibility

Quantified data were analyzed using Prism 9 (GraphPad Software). All bar graphs in the figures represent the mean ± s.d. from multiple determinations. When analyzing groups of data, the results are represented as SuperPlots^77^ showing the individual data points in different colors, the mean from each experiment and the mean ± s.d. of the means. Each experiment was replicated at least three times on different days. The statistical significance of differences between two conditions was calculated using Student’s *t*-test. The statistical significance for groups was calculated by one-way ANOVA with multiple comparisons using Dunnett’s or Tukey’s test as indicated in the figure legends. Significance is denoted using asterisks: *P < 0.05, **P < 0.01 and ***P < 0.001. *P* > 0.05 is not significant (NS). The total number of samples (*n*) analyzed in each experiment is indicated in the figure legends.

No statistical methods were used to predetermine sample sizes, but our sample sizes are similar to those reported in previous publications^15,16^. Instead, multiple independent experiments were carried out using several sample replicates as detailed in the figure legends. Data collection was not randomized. Data distribution was assumed to be normal, but this was not formally tested.

Data collection and analysis were not performed blinded to the conditions of the experiments.

### Reporting summary

Further information on research design is available in the Nature Portfolio Reporting Summary linked to this article.

## Online content

Any methods, additional references, Nature Portfolio reporting summaries, source data, extended data, supplementary information, acknowledgements, peer review information; details of author contributions and competing interests; and statements of data and code availability are available at 10.1038/s41593-024-01619-1.

### Supplementary information

Supplementary Information Supplementary Table 3 and Data 1 and 4.

Reporting Summary

Supplementary Table 1 Gene expression changes in RNA-seq analysis, related to Figs. 2 and 3. Columns indicate Ensembl gene IDs (gene), mean normalized read counts across all replicates in all conditions (baseMean), magnitude of differential expression in log_2_ scale (log_2_ fold change), standard errors of the log_2_ fold change estimate (lfcSE), Wald test statistic (stat), raw *P* values (pvalue), FDR (padj), gene symbols (symbol), UniProt protein IDs (uniprot) and aliases for gene symbols (alias), respectively. NAs given to the log_2_ fold changes, *P* values or padj suggest genes with zero counts in all samples (NAs in log_2_ fold change, pvalue and padj), genes with at least one replicate being outlier and removed from analysis (NAs in pvalue and padj) or uninformative genes being removed from the analysis (NAs in padj).

Supplementary Table 2 Gene expression changes in selected genes obtained from RNA-seq analysis, related to Figs. 2 and 3 and Extended Data Fig. 4. Genes represented in the MA plots and Venn diagrams (Figs. 2g and 3a,b and Extended Data Fig. 3a,b) were selected from Table 1 and collected in this table. Two different comparisons between two groups of genes are shown. The group name is indicated in the table title and the page tab. For each comparison, columns indicate Ensembl gene IDs (gene), mean normalized read counts across all replicates in all conditions (baseMean), magnitude of differential expression in fold changes (fold change), raw *P* values (pvalue), FDR (padj), gene symbols (symbol), UniProt protein IDs (uniprot) and aliases for gene symbols (alias), respectively.

Supplementary Video 1 Co-movement of *RPS7* mRNA with LAMP1-positive organelles in the axon of WT i3Neurons, related to Fig. 4. One-minute video analysis of particle movement in the axon from WT i3Neurons stably co-transfected with plasmids encoding HaloTag fused to PP7 coat protein and *RPS7* fused to 24 PP7 RNA stem-loop repeats, cultured for 25 d on glass coverslips and transiently transduced with a lentivirus encoding LAMP1-mNeonGreen. This video corresponds to the kymograph shown in Fig. 4b. Note that this video does not allow assignment of the movement to anterograde or retrograde transport because of the random orientation of axons on the coverslips.

Supplementary Video 2 Co-movement of *RPS27A* mRNA with LAMP1-positive organelles in the axon of WT i3Neurons, related to Fig. 4. One-minute video analysis of particle movement in the axon from WT i3Neurons stably co-transfected with plasmids encoding HaloTag fused to PP7 coat protein and *RPS27A* fused to 24 PP7 RNA stem-loop repeats, cultured for 25 d on glass coverslips and transiently transduced with a lentivirus encoding LAMP1-mNeonGreen. This video corresponds to the kymograph shown in Fig. 4d. Note that this video does not allow assignment of the movement to anterograde or retrograde transport because of the random orientation of axons on the coverslips.

Supplementary Data 2 Schematics of the first glass chrome mask, related to Fig. 2 and Extended Data Fig. 3. AutoCAD software was used to design the schematics for the microgrooves pattern of microfluidic devices. The scheme was used to generate the first glass chrome mask for the first layer of photolithographic patterning.

Supplementary Data 3 Schematics of the second glass chrome mask, related to Fig. 2 and Extended Data Fig. 3. AutoCAD software was used to design the schematics for the reservoirs pattern of microfluidic devices. The scheme was used to generate the second glass chrome mask for the second layer of photolithographic patterning.

### Source data

Source Data Fig. 1 Unprocessed western blots.

Source Data Fig. 2 Unprocessed western blots.

Source Data Fig. 6 Unprocessed western blots.

## Data Availability

Reagents generated in this study are available upon reasonable request. All data are available in the main text or Supplementary Information. Further information and requests for resources and reagents should be directed to the corresponding author. Bulk RNA-seq data have been deposited at GSE225479 and are publicly available as of the date of publication. Accession numbers are listed in the key resources table. Microscopy data reported in this paper will be shared by the lead contact upon reasonable request. Source data are provided with this paper.

## Code availability

This paper does not report original code. Any additional information required to reanalyze the data reported in this paper is available from the corresponding author upon reasonable request.
