# Supplementary material for: Messenger RNA transport on lysosomal vesicles maintains axonal mitochondrial homeostasis and prevents axonal degeneration
Source: Nat Neurosci. 2024 Apr 10;27(6):1087–102. doi: 10.1038/s41593-024-01619-1 (PMC11156585; doi:10.1038/s41593-024-01619-1)
Supplement: Supplementary file 1 — Supplementary Table 3 and Data 1 and 4. [file 41593_2024_1619_MOESM1_ESM.pdf]

nature neuroscience

Article

https://doi.org/10.1038/s41593-024-01619-1

Messenger RNA transport on lysosomal vesicles maintains axonal mitochondrial homeostasis and prevents axonal degeneration

In the format provided by the authors and unedited

Supplementary Information

Supplementary Data 1 Additional Materials and Methods

Microfluidic devices preparation. Related to Fig. 2 and Extended Data Fig. 3

Master mold fabrication: The master mold was made using standard photolithographic techniques. Briefly, a 4″ silicon wafer was dehydrated at 200°C for 20 min. To pattern the microgrooves, SU-8 2005 (Kayaku Advanced Materials) was spin-coated (Laurell Technologies) on the wafer for a desired height of 4 µm. The wafer was baked at 95°C for 2 min before being UV-exposed through the first glass-chrome mask (Front Range Photomask) (Supplementary Data 2) using a contact mask aligner (OAI Instruments). The wafer was baked post-exposure at 95°C for 3 min. To achieve a 100-µm tall reservoir, SU-EX 100 (DJ Microlaminates) dry-film resist was laminated over the wafer using a heated roll laminator (Pro-Lam PL1200HP) with a controlled speed of 1 ft/min and a roller temperature of 65°C. Following lamination, the wafer was baked at 65°C for 5 min and transferred to 95°C for 15 min. The wafer was then UV-exposed through the second glass-chrome mask (Supplementary Data 3) and baked at 65°C for 5 min. The temperature was ramped to 95°C and baked for an additional 10 min before gradually decreasing the temperature back to room temperature. The wafer was developed in a SU-8 developer bath (Kayaku Advanced Materials) with gentle agitation for 10-15 min. To increase durability, the master mold was hard-baked at 95°C for 15 min before allowing it to cool down to room temperature at a rate of 150°C/h. The wafer was silanized with tridecafluoro-1,1,2,2- tetrahydrooctyl-1-trichlorosilane (UCT Specialties) vapor for a minimum of 2 h to facilitate the unmolding steps. The final device structure is shown in Extended Data Fig. 2a,b.

Soft lithography for PDMS mold fabrication: Polydimethylsiloxane (PDMS) (Sylgard 184, Dow Corning) base and curing agent were mixed in a 10:1 (w:w) ratio in a planetary mixer (Thinky). The pre-mixed PDMS was poured onto the master mold, degassed, and cured at 80°C for 1 h. The cured PDMS was then unmolded and cut. The extreme ends of each reservoir were punched using a 3-mm round biopsy punch (Acuderm), the holes were connected with a single-edge carbon steel razor blade, taking care to leave a little of the 100-µm reservoir layer behind, in order to ensure the microgrooves had well-defined and reproducible lengths. The individual devices were cleaned with tape and plasma bonded to a PDMS slab, centered in a PDMS frame with an opening of approximately 2.25″x1.25″x0.5″ (LxWxH) to create the PDMS molds with incorporated open reservoirs. The PDMS molds were treated with oxygen plasma for 45 s and

exposed to tridecafluoro-1,1,2,2- tetrahydrooctyl-1-trichlorosilane vapor overnight to minimize adhesion.

Fabricating the epoxy master mold: Epoxy molds were prepared using a high-temperature two-part epoxy (301-2, Epoxy Technology) mixed as directed (100:35 (w:w) ratio of parts A and B). To ensure complete mixing, a three-stage mixing protocol was used: first a 4-min mixing and degassing steps in the planetary mixer, followed by mixing by hand for 2 min using a glass stirrer, and finally another mix/degas cycle in the planetary mixer. About 10-12 g of mixed epoxy was poured onto the silanized PDMS molds and then degassed under vacuum for 90 min. The epoxy was cured at 40°C for one hour, followed by 60°C for one hour, and finally 80°C for 3 hours, for a total of 5 hours of curing. The cure process is exothermic, and we found that for the volume of epoxy used, the gradual temperature increase was necessary to avoid substantial overshoot of the desired 80°C final temperature. After curing, the epoxy was unmolded from the PDMS molds, washed with isopropanol, dried with nitrogen gas and treated with oxygen plasma for 45 s before silanizing overnight with tridecafluoro-1,1,2,2-tetrahydrooctyl-1-trichlorosilane vapor.

Final device fabrication: The resulting epoxy master molds were used to cast the final microfluidic chambers using PDMS base and curing agent mixed in a 10:1 (w:w) ratio in a planetary mixer. The mixed PDMS was poured onto the epoxy master mold, degassed, and cured at 80°C for 1 h. The cured PDMS was then unmolded and trimmed. The individual devices were cleaned with tape, treated with oxygen plasma for 45 s and bonded to a 24x50-mm acid-etched coverslip (Daigger). This final device was then coated with polylysine and laminin.

Mouse husbandry. Related to Fig. 6 and 7 and Extended Data Fig. 8 and 10

All mouse procedures were conducted following the NIH Guide for the Care and Use of Laboratory Animals, under protocol #21-021 approved by the NICHD Animal Care and Use Committee. Mice used in the study were naïve and had normal health status. For timed pregnancies, we housed single male and female mice of 6 weeks of age in separate cages under a 12-h light-dark cycle. Housing conditions were maintained at ~23°C with 40-60% humidity. To time the deliveries, the female's weight was recorded, and the female was transferred overnight into the cage housing the male for no longer than 12 h. The following day, the two animals were separated, and the female was checked for the presence of plaque. If the plaque was absent, the female was transferred again to the male cage the following night. We monitored the weight of the female to determine increases over time. When the pregnancy was successful, we harvested

embryos after 17 days for culture of primary cortical neurons. Neurons of mice with identical genotype and from the same litter were pooled and analyzed.

SDS-PAGE and immunoblotting. Related to Fig. 1, 2 and 6

i3Neurons were grown in the microfluidic devices for 45 days. Cells were washed twice with ice-cold PBS. Proteins from three axonal or neuronal compartments were extracted in a total of 100 µl 1X Laemmli sample buffer (1x LSB) (Bio-Rad) supplemented with 2.5% v/v 2-mercaptoethanol (Sigma-Aldrich), heated at 95°C for 5 min, and resolved by SDS-PAGE. iPSCs were grown in a Matrigel-coated 3.5 cm plate for 24 h and then extracted with 500 µl of 1x LSB supplemented with 2.5% v/v 2-mercaptoethanol, heated at 95°C for 5 min, and resolved by SDS-PAGE. Gels were blotted onto nitrocellulose membrane and blocked with 5% w/v non-fat milk in Tris-buffered saline, 0.1% v/v Tween 20 (TBS-T) for 20 min. Membranes were sequentially incubated with primary antibody and secondary HRP-conjugated antibody diluted in TBS-T. SuperSignal West Dura Reagents (Thermo Scientific) were used for detection of the antibody signal with a Bio-Rad ChemiDoc MP imaging system.

Puromycylation proximity ligation assay (Puro-PLA). Related to Fig. 5

WT, KO and rescue i3Neurons were grown on coverslips for 25 days. Neurons were then incubated with 2 µM puromycin in complete BrainPhys medium for 10 min. Neurons were washed twice with ice-cold 1X PBS and fixed with 4% w/v paraformaldehyde (Electron Microscopy Sciences) in PBS for 30 min. As a negative control, one batch of WT i3Neurons was fixed without puromycin treatment. After fixation, i3Neurons were washed three times with PBS and blocked with Duolink® Blocking Solution (#DUO82007, Sigma Aldrich) for 30 min at 37°C in a humidified chamber. Primary antibodies were diluted in Duolink® Antibody Diluent and incubated with the i3Neurons for 30 min at 37°C. Coverslips were washed twice with 1x wash buffer A (5 min each) at room temperature. Duolink® PLUS (#DUO92002, Sigma Aldrich) and Duolink® MINUS PLA (#DUO92004, Sigma Aldrich) secondary antibody probes were diluted (1:5) in the Duolink® Antibody Diluent and applied to neurons for 1 h at 37°C. The DNA probes were washed with 1x wash buffer A (5 min each) at room temperature and further developed following the protocol of Duolink® in situ detection Reagents Red (#DUO92008, Sigma Aldrich). Briefly, the samples were ligated using DNA Ligase (DUO82027, Sigma Aldrich) mixed in 1x ligation buffer (DUO82009, Sigma Aldrich) for 30 min at 37°C. i3Neurons were washed with 1x wash buffer A at room temperature and incubated with DNA polymerase (#DUO82028, Sigma Aldrich) in 1x amplification buffer (DUO82011, Sigma Aldrich) for 100 min at 37°C. Neurons were then washed with wash buffer B twice for 10 min each at room

temperature. Neurons were further fixed with 4% paraformaldehyde for 10 min and washed with 1x PBS. Neurons were finally immunolabelled with anti-MAP2 antibody following the immunofluorescence protocol described above.

Detection of mRNA using RNAScope *in situ* hybridization (ISH). Related to Extended Data Fig. 2

To examine the localization of *RPL41* mRNA, we used the RNAScope Multiplex Fluorescent v2 assay kit (ACD) according to the manufacturer's instructions. Briefly, WT, BORCS5-KO, BORCS5-rescue i3Neurons were grown on coverslips for 20 days. Neurons were then fixed with 4% paraformaldehyde for 30 min, dehydrated through a series of increasing concentrations of ethanol (50%, 70%, 2x 100% anhydrous) and rehydrated (70%, 50%, 1xPBS) for 1 min per step. Neurons were then treated with 0.1% Tween-20, followed by treatment with H_2O_2 and protease. A custom-made C1 RNAscope Target Probe for *RPL41* was used to hybridize the neurons, followed by amplification and HRP C1 detection of the probe. Cells were then analyzed by immunofluorescence microscopy with an antibody to the axonal marker NFH.

Electron microscopy. Related to Fig. 6 and 7 and Extended Data Fig. 8 and 10

iPSC-derived i3Neurons were grown for 25 days on 18-mm glass coverslips as described before, and fixed in 2.5% glutaraldehyde, 2% formaldehyde, 2 mM $CaCl_2$ in 0.1 M cacodylate buffer pH 7.4 for 15 min at room temperature followed by 45 min on ice. The fixative was prepared at two-fold concentration and added to an equal volume of medium directly to the cultured neurons to prevent stress due to removal of the medium. Coverslips were washed 4 times for 5 min and post-fixed 30 min in fixation buffer supplemented with 0.5% OsO_4 and 0.5% potassium ferrocyanide at 4°C. Alternatively, brains were harvested from E17 WT and BORCS5-KO mouse embryos and fixed in 2.5% glutaraldehyde, 2% formaldehyde, 2 mM $CaCl_2$ in 0.1 M cacodylate buffer pH 7.4 for 2 h on ice. Brains were washed 4 times for 5 min and post-fixed 30 min in fixation buffer supplemented with 0.5% OsO_4 and 0.5% potassium ferrocyanide at 4°C. Samples were extensively washed with the same buffer, stained for 30 min (coverslips) or 90 min (brains) with 1% tannic acid followed by 30 min (coverslips) or overnight (brains) with 2% uranyl acetate in 50 mM acetate pH 5.5. Samples were dehydrated through a series of increasing concentrations of ethanol (50%, 70%, 90%, 3x100% anhydrous) and embedded in EMBed 812 epoxy resin (EMS). The resin was then polymerized for 60 h at 65°C. For cultured neurons, the coverslips were dissolved with hydrofluoric acid, regions of interest containing cells identified by phase contrast microscopy, cut out, and remounted. Serial sections 90-nm thick were cut parallel to the plane of the coverslip. Blocks containing the brain samples were trimmed, semi-

thin sections of 300 nm were cut and stained with epoxy stain (EMS) to find the section location within the sample under a light microscope. Once confirmed, 90 nm sections were cut. Sections from both types of samples were mounted on formvar/carbon-coated slot (0.5x2 mm) EM grids (EMS) and post-stained with lead citrate. Sections were imaged in a FEI Tecnai 20 transmission electron microscope operated at 120 kV. Images were recorded on an AMT XR81 wide field CCD camera. Axons were morphologically identified by size (thin processes, with little cytoplasm, away from the soma), and the presence of synaptic vesicles and neurofilaments.

Quantification of mitochondria size. Related to Fig. 6

WT, BORCS5-KO and BORCS7-KO i3Neurons were grown on microfluidic devices for 25 days. Cells were fixed and immunostained for TOMM20 as described above. Images of axonal mitochondria were taken using a Zeiss LSM880 confocal microscope with a Plan Apochromat 63x objective (N.A. 1.40). Mitochondrial average size and length were measured by Fiji (https://fiji.sc/). Z-stack images were converted to maximum intensity projections (MIP) and fluorescence intensity was used to threshold the individual mitochondria in an image field. The average size and length were obtained using the 'analyze particle' tab. Statistics of mitochondrial measurements were obtained from ~12 fields of axonal chambers from three different cultures.

Mouse cortical neuron transfection. Related to Fig. 8

Mouse cortical neurons were cultured on cover glasses for 3 days with neurobasal medium. Then, neurons were transfected with pEF-GFP (Addgene, Cat#11154) expressing cytosolic GFP under the control of the EF1-α promoter. The transfection was done using a calcium phosphate transfection kit (Takara, Cat#631312). For each transfection, a tube of 2 M calcium chloride, sterile water and 2 μg DNA was prepared. Then, 2X HEPES-buffered saline (HBS) was added dropwise to the previous tube with gentle vortexing. The transfection mix was incubated 15 min at room temperature and added to the cells with culture medium. Cells were incubated for 1.5 h at 37°C and 5% CO_2. Next, cells were washed three times with 1X PBS and kept with neurobasal medium in the incubator. At day 7, the neurons were fixed and analyzed.

Supplementary Data 2 AutoCAD file for the schematics of the first glass-chrome mask. Related to Fig. 2 and Extended Data Fig. 3

AutoCAD software was used to design the schematics for the microgrooves pattern of microfluidic devices. The scheme was used to generate the first glass-chrome mask for the first layer of photolithographic patterning.

Supplementary Data 3 AutoCAD file for the schematics of the second glass-chrome mask. Related to Fig. 2 and Extended Data Fig. 3

AutoCAD software was used to design the schematics for the reservoirs pattern of microfluidic devices. The scheme was used to generate the second glass-chrome mask for the second layer of photolithographic patterning.

Supplementary Data 4 Raw data of moving mRNA particles obtained from single kymographs. Related to Fig. 4

Absolute numbers for graphs c, e, and h in Fig. 4. Data represent raw numbers of co-moving, anterograde, retrograde, or static events in single kymographs for the indicated mRNA and cell line.

Supplementary Table 1 Excel file showing gene expression changes in RNA-seq analysis. Related to Fig. 2 and 3

Columns indicate Ensembl gene IDs (gene), mean normalized read counts across all replicates in all conditions (baseMean), magnitude of differential expression in log2 scale (log2FoldChange), standard errors of the log2 fold change estimate (lfcSE), Wald test statistic (stat), raw p-values (pvalue), FDR (padj), gene symbols (symbol), UniProt protein IDs (uniprot), and aliases for gene symbols (alias), respectively. NAs given to the log2FoldChanges, pvalues, or padj suggest genes with zero counts in all samples (NAs in log2FoldChange, pvalue, and padj), genes with at least one replicate being outlier and removed from analysis (NAs in pvalue and padj), or uninformative genes being removed from the analysis (NAs in padj).

Supplementary Table 2 Excel file showing gene expression changes in selected genes obtained from RNA-seq analysis. Related to Fig. 2, 3 and Extended Data Fig. 4

Genes represented in the MA plots and Venn diagrams (Fig. 2g, 3a,b, and Extended Data Fig. 3a,b) were selected from Table 1 and collected in this table. Two different comparisons between two groups of genes are shown. The group name is indicated in the table title and the page tab. For each comparison, columns indicate Ensembl gene IDs (gene), mean normalized read counts across all replicates in all conditions (baseMean), magnitude of differential expression in fold changes (fold change), raw p-values (pvalue), FDR (padj), gene symbols (symbol), UniProt protein IDs (uniprot), and aliases for gene symbols (alias), respectively.

Supplementary Table 3 List of reagents and resources used in this study

Source and identifier are indicated. When available, RRID identifier is also indicated.

Supplementary Video 1 Co-movement of *RPS7* mRNA with LAMP1-positive organelles in the axon of WT i3Neurons. Related to Fig. 4

One-minute video analysis of particle movement in the axon from WT i3Neurons stably co-transfected with plasmids encoding HaloTag fused to PP7 coat protein and *RPS7* fused to 24 PP7 RNA stem-loop repeats, cultured for 25 days on glass coverslips, and transiently transduced with a lentivirus encoding LAMP1-mNeonGreen. This video corresponds to the kymograph shown in Fig. 4b. Note that this video does not allow assignment of the movement to anterograde or retrograde transport because of the random orientation of axons on the coverslips.

Supplementary Video 2 Co-movement of *RPS27A* mRNA with LAMP1-positive organelles in the axon of WT i3Neurons. Related to Fig. 4

One-minute video analysis of particle movement in the axon from WT i3Neurons stably co-transfected with plasmids encoding HaloTag fused to PP7 coat protein and *RPS27A* fused to 24 PP7 RNA stem-loop repeats, cultured for 25 days on glass coverslips, and transiently transduced with a lentivirus encoding LAMP1-mNeonGreen. This video corresponds to the kymograph shown in Fig. 4d. Note that this video does not allow assignment of the movement to anterograde or retrograde transport because of the random orientation of axons on the coverslips.

Supplementary Data 4

Fig. 4c RPS7

WT

Co-moving	Independent movement	TOTAL	co-moving %
2	0	2	100
1	0	1	100
1	1	2	50
2	0	2	100
1	0	1	100
2	0	2	100
2	1	3	66.7
1	0	1	100
1	0	1	100
2	0	2	100
1	0	1	100
1	0	1	100
1	0	1	100
1	0	1	100
1	0	1	100
1	0	1	100
1	0	1	100
1	0	1	100
1	0	1	100
1	0	1	100
1	0	1	100
1	0	1	100

BORCS5 KO

Co-moving	Independent movement	TOTAL	co-moving %
0	0	0	0
0	0	0	0
0	0	0	0
0	0	0	0
0	0	0	0
0	0	0	0
0	0	0	0
0	0	0	0
0	0	0	0
0	0	0	0
0	0	0	0
0	0	0	0
0	0	0	0
0	0	0	0
0	0	0	0
0	0	0	0
0	0	0	0
0	0	0	0
0	0	0	0
0	0	0	0
0	0	0	0
0	0	0	0

BORCS7 KO

Co-moving	Independent movement	TOTAL	co-moving %
0	0	0	0
0	0	0	0
0	0	0	0
0	0	0	0
0	0	0	0
0	0	0	0
0	0	0	0
0	0	0	0
0	0	0	0
0	0	0	0
0	0	0	0
0	0	0	0
0	0	0	0
0	0	0	0
0	0	0	0
0	0	0	0
0	0	0	0
0	0	0	0
0	0	0	0
0	0	0	0
0	0	0	0
0	0	0	0

Fig. 4e RPS 27A

WT

Co-moving	Independent movement	TOTAL	co-moving %
1	0	1	100
3	0	3	100
3	1	4	75
6	2	8	75
1	1	2	50
1	0	1	100
1	0	1	100
1	0	1	100
3	1	4	75
1	0	1	100
1	0	1	100
2	0	2	100
7	0	7	100
1	0	1	100
1	0	1	100
1	0	1	100
2	0	2	100
1	0	1	100
1	0	1	100
1	0	1	100
1	0	1	100
1	0	1	100

BORCS5 KO

Co-moving	Independent movement	TOTAL	co-moving %
0	0	0	0
0	0	0	0
0	0	0	0
0	0	0	0
0	0	0	0
0	0	0	0
0	0	0	0
0	0	0	0
0	0	0	0
0	0	0	0
0	0	0	0
0	0	0	0
0	0	0	0
0	0	0	0
0	0	0	0
0	0	0	0
0	0	0	0
0	0	0	0
0	0	0	0
0	0	0	0
0	0	0	0
0	0	0	0

BORCS7 KO

Co-moving	Independent movement	TOTAL	co-moving %
0	0	0	0
0	0	0	0
0	0	0	0
0	0	0	0
0	0	0	0
0	0	0	0
0	0	0	0
0	0	0	0
0	0	0	0
0	0	0	0
0	0	0	0
0	0	0	0
0	0	0	0
0	0	0	0
0	0	0	0
0	0	0	0
0	0	0	0
0	0	0	0
0	0	0	0
0	0	0	0
0	0	0	0
0	0	0	0

Fig. 4h RPS7

WT				%		
Antero	Retro	Static	TOT	Antero	Retro	Static
0	1	0	1	0	100	0
1	0	0	1	100	0	0
1	0	1	2	50	0	50
1	1	0	2	50	50	0
2	1	0	3	67	33	0
1	0	0	1	100	0	0
0	1	0	1	0	100	0
1	1	0	2	50	50	0
0	2	0	2	0	100	0
2	0	2	4	50	0	50
0	1	0	1	0	100	0
1	1	0	2	50	50	0
0	2	0	2	0	100	0
1	0	0	1	100	0	0
0	1	0	1	0	100	0
0	1	0	1	0	100	0
0	1	0	1	0	100	0
0	1	1	2	0	50	50
0	1	0	1	0	100	0
0	1	3	4	0	25	75
1	0	0	1	100	0	0
2	0	0	2	100	0	0
0	2	0	2	0	100	0
0	1	0	1	0	100	0

BORCS5 KO				%		
Antero	Retro	Static	TOT	Antero	Retro	Static
0	0	0	0	0	0	0
0	0	0	0	0	0	0
0	0	0	0	0	0	0
0	0	0	0	0	0	0
0	0	0	0	0	0	0
0	0	0	0	0	0	0
0	0	0	0	0	0	0
0	0	0	0	0	0	0
0	0	0	0	0	0	0
0	0	0	0	0	0	0
0	0	0	0	0	0	0
0	0	0	0	0	0	0
0	0	0	0	0	0	0
0	0	0	0	0	0	0
0	0	0	0	0	0	0
0	0	0	0	0	0	0
0	0	0	0	0	0	0
0	0	0	0	0	0	0
0	0	0	0	0	0	0
0	0	0	0	0	0	0
0	0	0	0	0	0	0
0	0	0	0	0	0	0
0	0	0	0	0	0	0
0	0	0	0	0	0	0

BORCS7 KO				%		
Antero	Retro	Static	TOT	Antero	Retro	Static
0	0	0	0	0	0	0
0	0	0	0	0	0	0
0	0	0	0	0	0	0
0	0	0	0	0	0	0
0	0	0	0	0	0	0
0	0	0	0	0	0	0
0	0	0	0	0	0	0
0	0	0	0	0	0	0
0	0	0	0	0	0	0
0	0	0	0	0	0	0
0	0	0	0	0	0	0
0	0	0	0	0	0	0
0	0	0	0	0	0	0
0	0	0	0	0	0	0
0	0	0	0	0	0	0
0	0	0	0	0	0	0
0	0	0	0	0	0	0
0	0	0	0	0	0	0
0	0	0	0	0	0	0
0	0	0	0	0	0	0
0	0	0	0	0	0	0
0	0	0	0	0	0	0
0	0	0	0	0	0	0
0	0	0	0	0	0	0

Fig. 4h RPS27A

WT				WT%		
Anterc	Retro	Static	TOT	Antero	Retro	Static
0	1	0	1	0	100	0
1	0	0	1	100	0	0
0	1	0	1	0	100	0
0	2	0	2	0	100	0
0	1	0	1	0	100	0
0	1	2	3	0	33	67
1	2	0	3	33	67	0
1	1	1	3	33	33	33
1	1	0	2	50	50	0
0	2	1	3	0	67	33
1	0	0	1	100	0	0
0	1	0	1	0	100	0
0	2	3	5	0	40	60
0	1	0	1	0	100	0
0	2	0	2	0	100	0
0	1	0	1	0	100	0
0	1	0	1	0	100	0
3	2	1	6	50	33	17
0	1	0	1	0	100	0
0	1	0	1	0	100	0
0	1	1	2	0	50	50

BORCS5 KO				%		
Antero	Retro	Static	TOT	Antero	Retro	Static
0	0	0	0	0	0	0
0	0	0	0	0	0	0
0	0	0	0	0	0	0
0	0	0	0	0	0	0
0	0	0	0	0	0	0
0	0	0	0	0	0	0
0	0	0	0	0	0	0
0	0	0	0	0	0	0
0	0	0	0	0	0	0
0	0	0	0	0	0	0
0	0	0	0	0	0	0
0	0	0	0	0	0	0
0	0	0	0	0	0	0
0	0	0	0	0	0	0
0	0	0	0	0	0	0
0	0	0	0	0	0	0
0	0	0	0	0	0	0
0	0	0	0	0	0	0
0	0	0	0	0	0	0
0	0	0	0	0	0	0
0	0	0	0	0	0	0
0	0	0	0	0	0	0
0	0	0	0	0	0	0
0	0	0	0	0	0	0

BORCS7 KO				%		
Anterc	Retro	Static	TOT	Antero	Retro	Static
0	0	0	0	0	0	0
0	0	0	0	0	0	0
0	0	0	0	0	0	0
0	0	0	0	0	0	0
0	0	0	0	0	0	0
0	0	0	0	0	0	0
0	0	0	0	0	0	0
0	0	0	0	0	0	0
0	0	0	0	0	0	0
0	0	0	0	0	0	0
0	0	0	0	0	0	0
0	0	0	0	0	0	0
0	0	0	0	0	0	0
0	0	0	0	0	0	0
0	0	0	0	0	0	0
0	0	0	0	0	0	0
0	0	0	0	0	0	0
0	0	0	0	0	0	0
0	0	0	0	0	0	0
0	0	0	0	0	0	0
0	0	0	0	0	0	0
0	0	0	0	0	0	0
0	0	0	0	0	0	0
0	0	0	0	0	0	0

Supplementary Table 3

REAGENT or RESOURCE	SOURCE	IDENTIFIER
Antibodies		
BORCS5 (LOH12CR1), rabbit, used 1:500 for IB	Proteintech	Cat#17169-1-AP, RRID:AB_2137150
BORCS7 (C10orf32), rabbit, used 1:500 for IB	Abnova	Cat#PAB23142, RRID:AB_11122571
GAPDH (0411) HRP conjugated, used 1:1,000 for IB	Santa Cruz	Cat#sc-47724, RRID:AB_627678
Alpha-tubulin, mouse, used 1:5,000 for IB	Santa Cruz	Cat#sc-32293 RRID:AB_628412
LAMP1, mouse, human reactivity, used 1:500 for IF	Developmental Studies Hybridoma Bank	Cat#H4A3, RRID:AB_2296838
LAMP1, rat, mouse reactivity, used 1:500 for IB, 1:500 for IF	Developmental Studies Hybridoma Bank	Cat#1D4B, RRID:AB_2134500
MAP2, chicken, used 1:1,000 for IB, 1:500 for IF	Abcam	Cat#ab5392, RRID:AB_2138153
MAP2 (H-300), rabbit, used 1:500 for IF	Santa Cruz	Cat#sc-20172, RRID:AB_2250101
Synaptophysin 1 (SYP1) (D-4), mouse, used 1:1,000 for IB, 1:500 for IF	Santa Cruz	Cat#sc-17750, RRID:AB_628311
LAMTOR4 (C7orf59) (D4P6O), rabbit, used 1:500 for IF	Cell Signaling	Cat#13140, RRID:AB_2798129
Synaptic vesicle glycoprotein 2A (SV2), mouse, used 1:200 for IB, 1:200 for IF, 1:500 for IHC	Developmental Studies Hybridoma Bank	Cat#SV2, RRID:AB_2315387
TOMM20, rabbit, used 1:500 for IF, 1:200 for IB	Proteintech	Cat#11802-1-AP, RRID:AB_2207530
Tau-1, clone PC1C6, human reactivity, mouse, used 1:500 for IF	Millipore Sigma	Cat#MAB3420
Tau, guinea pig, mouse reactivity, used 1:200 for IF	Synaptic Systems	Cat#314 004, RRID:AB_1547385
Ankyrin-G, mouse, used 1:3 for IF	NeuroMab	Cat#73-146, RRID:AB_10697718
NDUFS1 (E4K3E), rabbit, used 1:100 for IB	Cell Signaling	Cat#70264
SDHA, mouse, used 1:100 for IB	Abcam	Cat#ab14715, RRID:AB_301433
CYCS (D18C7), rabbit, used 1:100 for IB	Cell Signaling	Cat#11940, RRID:AB_2637071
CYCS (6H2.B4), mouse, used 1:200 for IF	BD Bioscience	Cat#556432, RRID:AB_396416
COX IV (3E11), rabbit, used 1:100 for IB	Cell Signaling	Cat#4850, RRID:AB_2085424
ATP5A (15H4C4), mouse, used 1:100 for IB	Abcam	Cat#ab14748, RRID:AB_301447
MIC60/Mitofilin, rabbit, used 1:100 for IB	Proteintech	Cat#10179-1-AP, RRID:AB_2127193
MIC10/MINOS1, rabbit, used 1:100 for IB	Novus Biologicals	Cat#NBP1-91587, RRID:AB_11030043
LC3 (D11), rabbit, human reactivity, used 1:200 for IF	Cell Signaling	Cat#3868, RRID:AB_2137707
LC3A/B (D3U4C), rabbit, mouse reactivity, used 1:200 for IF	Cell Signaling	Cat#12741, RRID:AB_2617131
TGN46, sheep, used 1:1,000 for IF	Bio-Rad	Cat#AHP500G, RRID:AB_323104
Synapsin 1, rabbit, used 1:1,000 for IB	Thermo Fisher Scientific	Cat#PA1-4673, RRID:AB_561585

M6PR-CI (2G11), mouse, used 1:200 for IF	Abcam	Cat# ab2733, RRID:AB_2122792
EEA1(C45B10) rabbit, used 1:500 for IF	Cell Signaling	Cat#3288, RRID:AB_2096811
Neurofilament heavy polypeptide, chicken, used 1:1,000 for IF	Abcam	Cat#ab4680 RRID:AB_304560
RPL24, rabbit, used 1:50 for IF	Proteintech	Cat# 17082-1-AP RRID:AB_2181728
RPS27A, rabbit, used 1:50 for IF	MyBioSource	Cat#MBS7103451
Puromycin (12D10), mouse, 1:500 for IF	Millipore Sigma	Cat# MABE343 RRID:AB_2566826
Alexa Fluor 647-conjugated goat anti chicken IgY, used 1:1,000 for IF	Thermo Fisher Scientific	Cat#A-21449, RRID:AB_1500594
Alexa Fluor 555-conjugated donkey anti mouse IgG, used 1:1,000 for IF	Thermo Fisher Scientific	Cat#A-31570, RRID:AB_2536180
Alexa Fluor 488-conjugated goat anti rat IgG, used 1:1,000 for IF	Thermo Fisher Scientific	Cat#A-11006 RRID:AB_141373
Alexa Fluor 488-conjugated donkey anti rabbit IgG, used 1:1,000 for IF	Thermo Fisher Scientific	Cat#A-21206, RRID:AB_2535792
Alexa Fluor 647-conjugated goat anti sheep IgG, used 1:1,000 for IF	Thermo Fisher Scientific	Cat#A-21448, RRID:AB_2535865
Alexa Fluor 488-conjugated goat anti guinea pig IgG, used 1:1,000 for IF	Thermo Fisher Scientific	Cat#A-11073 RRID:AB_2534117
HRP-conjugated goat anti-rabbit IgG (H+L), used 1:5,000 for IF	Jackson ImmunoResearch	Cat#111-035-144, RRID:AB_2307391
HRP-conjugated goat anti-chicken IgY (H+L), used 1:5,000 for IF	Jackson ImmunoResearch	Cat#103-035-155, RRID:AB_2337381
HRP-conjugated donkey anti-mouse IgG (H+L), used 1:5,000 for IF	Jackson ImmunoResearch	Cat#715-035-150, RRID:AB_2340770
Chemicals, peptides, and recombinant proteins		
Lipofectamine 2000	Thermo Fisher Scientific	Cat#11668019
Lipofectamine 3000	Thermo Fisher Scientific	Cat#L3000001
SPY650-Tubulin	Cytoskeleton Inc	Cat#CY-SC503
MitoTracker™ Green FM	Thermo Fisher Scientific	Cat#M7514
Halo substrate JF646	Promega	Cat#GA1120
FCCP	Abcam	Cat#ab120081
Rotenone	Abcam	Cat#ab143145
TMRE	Thermo Fisher Scientific	Cat#T669
MitoSOX	Thermo Fisher Scientific	Cat#M36008
Puromycin	Sigma-Aldrich	Cat#P9620
Critical commercial assays		
Duolink In situ Detection Reagent Red	Sigma-Aldrich	Cat#DUO92008
Duolink In situ PLA probe Anti-Mouse Minus	Sigma-Aldrich	Cat#DUO92004
Duolink In situ PLA probe Anti-Rabbit Plus	Sigma-Aldrich	Cat#DUO92002
Calcium phosphate transfection kit	Takara	Cat#631312
RNAscope Multiplex Fluorescent Reagent Kit v2	ACD Bio	Cat#323110
Made-to-Order C1 RNAscope Target Probe for RPL41	ACD Bio	Cat#474811
RNAscope Probe Diluent	ACD Bio	Cat#300041
RNAscope H_2O_2 and Protease Reagents	ACD Bio	Cat#322381

Deposited data		
Gene Expression Omnibus (GEO)	GSE225479	https://www.ncbi.nlm.nih.gov/geo/query/acc.cgi?acc=GSE225479
Experimental models: cell lines		
Induced pluripotent stem cells (iPSCs) expressing the neuronal transcriptional activator neurogenin 2 (NGN2) under the control of a doxycycline-inducible promoter	Refs [42,43]	N/A
iPSCs BORCS5-KO	This study	N/A
iPSCs BORCS7-KO	This study	N/A
iPSCs BORCS5-rescued cells	This study	N/A
iPSCs BORCS7-rescued cells	This study	N/A
Primary hippocampal cultures from WT and BORCS5-KO mouse E17 brains	This study	N/A
HEK 293T/17	ATCC	Cat# CRL-11268
Experimental models: organisms/strains		
Mice: BORCS5-KO 10 bp deletion	Ref [11]	N/A
Mice: C57BL/6J	The Jackson Laboratory	Stock No: 000664 | Black 6
Oligonucleotides		
sgRNA: BORCS5 Forward 5′-caccgCTCAGGGCTCCCAGGCCTCA-3'	Eurofins Genomics	N/A
sgRNA: BORCS5 Reverse 5′-aaacTGAGGCCTGGGAGCCCTGAGc -3'	Eurofins Genomics	N/A
Primer: Sequencing BORCS5 KO Forward 5′-AGTGACTCCTTCACCAGCCAAGCAT -3'	Eurofins Genomics	N/A
Primer: Sequencing BORCS5 KO Reverse 5′-GTAGAGGGAAAGTAGTAGGGCTACAC -3'	Eurofins Genomics	N/A
sgRNA: BORCS7 Forward 5′-caccgACGGAGAAGGTGACCACCTG -3'	Eurofins Genomics	N/A
sgRNA: BORCS7 Reverse 5′-aaacCAGGTGGTCACCTTCTCCGTc -3'	Eurofins Genomics	N/A
Primer: Sequencing BORCS7 KO Forward 5′-GGCCCCGGCGACTCACCATCGTCAG -3'	Eurofins Genomics	N/A
Primer: Sequencing BORCS7 KO Reverse 5′-TACAATTCCCAAGATGCAACGCGAC -3'	Eurofins Genomics	N/A
Primer: BORCS5 Forward 5′-TGGTTCATTgccacCATGGGCAGTGAGCAG -3'	Eurofins Genomics	N/A
Primer: BORCS5 Reverse 5′-AGCTCCGCTTCCCAGCCTGAGCTCG -3'	Eurofins Genomics	N/A
Primer: BORCS7-HA Forward 5′-TCATTGCCACCATGATGGCGACTGGAA -3'	Eurofins Genomics	N/A
Primer: BORCS7-HA Reverse 5′-CTCCGCTTCCAGCGTAGTCTGGGAC -3'	Eurofins Genomics	N/A
Primer: EF1alpha Forward 5′-TCTAGACCACGTGCCCGTCAGTGG -3′	Eurofins Genomics	N/A
Primer: EF1alpha Forward 5′-GGTGGCAATGAACCAAG -3′	Eurofins Genomics	N/A
Recombinant DNA		

Lentivirus packaging vector psPAX2	Gift from Didier Trono; Addgene plasmid # 12260; http://n2t.net/addgene:12260	Cat#12260, RRID:Addgene_12260
Lentivirus packaging vector pMD2.G	Gift from Didier Trono; Addgene plasmid # 12259; http://n2t.net/addgene:12259	Cat#12259, RRID:Addgene_12259
pAdVantage	Promega	E1711
px458	Gift from Feng Zhang; Addgene plasmid # 48138; http://n2t.net/addgene:48138	Cat#48138, RRID:Addgene_48138
EF1A-BORCS5-P2A-eGFP	Laboratory of Michael Ward	N/A
EF1A-BORCS5-G2>A-P2A-eGFP	Laboratory of Michael Ward	N/A
EF1A-BORCS7-HA-P2A-eGFP	Laboratory of Michael Ward	N/A
PB-EF1A-PP7 coat protein-HALO-GB1x3-WPRE	Laboratory of Michael Ward	N/A
PB-EF1A-SunTag-RPS7-PP7-WPRE	Laboratory of Michael Ward	N/A
PB-EF1A-RPS27A-UTR-PP7-WPRE	Laboratory of Michael Ward	N/A
Super PiggyBac Transposase	Laboratory of Michael Ward	N/A
PGK-hLAMP1-mNeonGreen	Laboratory of Michael Ward	N/A
PGK-hLAMP1-3X KBS-mNeonGreen	This study	N/A
pEF-GFP	Gift from Connie Cepko; Addgene plasmid # 11154; https://www.addgene.org/11154/	Cat#11154
**Software and algorithms**		
Fiji/ImageJ	NIH	https://fiji.sc/ RRID:SCR_002285 v2.9.0
IMARIS	Oxford Instruments	https://imaris.oxinst.com/ RRID:SCR_007370 v8.2.0
GraphPad Prism	GraphPad	http://www.graphpad.com/ RRID:SCR_002798 v9.5.0
SnapGene	Dotmatics	http://www.snapgene.com/ RRID:SCR_015052 v6.2.1

BioRender	BioRender	https://www.biorender.com/<br>RRID:SCR_018361<br>v2023
AutoCAD	Autodesk	https://web.autocad.com/login<br>v2023
STAR	https://github.com/alexdobin/STAR	v2.7.8a
featureCounts (subread)	https://subread.sourceforge.net/	v2.0.1
R	https://www.r-project.org/	v4.0.3
DESeq2	https://bioconductor.org/packages/release/bioc/html/DESeq2.html	v1.30.1
ensembldb	https://bioconductor.org/packages/release/bioc/html/ensembldb.html	v2.14.0
clusterProfiler	https://bioconductor.org/packages/release/bioc/html/clusterProfiler.html	v3.18.1
GeneTonic	https://bioconductor.org/packages/release/bioc/html/GeneTonic.html	v1.5.2
Zen black	https://www.micro-shop.zeiss.com/en/us/softwarefinder/software-categories/zen-black/	v14.0
