# Supplementary material for: Messenger RNA transport on lysosomal vesicles maintains axonal mitochondrial homeostasis and prevents axonal degeneration
Source: Nat Neurosci. 2024 Apr 10;27(6):1087–102. doi: 10.1038/s41593-024-01619-1 (PMC11156585; doi:10.1038/s41593-024-01619-1)
Supplement: Supplementary file 2 — Reporting Summary [file 41593_2024_1619_MOESM2_ESM.pdf]

nature portfolio

Corresponding author(s): Juan S. Bonifacino

Last updated by author(s): Mar 21, 2024

Reporting Summary

Nature Portfolio wishes to improve the reproducibility of the work that we publish. This form provides structure for consistency and transparency in reporting. For further information on Nature Portfolio policies, see our Editorial Policies and the Editorial Policy Checklist.

Statistics

For all statistical analyses, confirm that the following items are present in the figure legend, table legend, main text, or Methods section.

n/a	Confirmed	
☐	☒	The exact sample size (*n*) for each experimental group/condition, given as a discrete number and unit of measurement
☐	☒	A statement on whether measurements were taken from distinct samples or whether the same sample was measured repeatedly
☐	☒	The statistical test(s) used AND whether they are one- or two-sided<br>*Only common tests should be described solely by name; describe more complex techniques in the Methods section.*
☒	☐	A description of all covariates tested
☐	☒	A description of any assumptions or corrections, such as tests of normality and adjustment for multiple comparisons
☐	☒	A full description of the statistical parameters including central tendency (e.g. means) or other basic estimates (e.g. regression coefficient) AND variation (e.g. standard deviation) or associated estimates of uncertainty (e.g. confidence intervals)
☐	☒	For null hypothesis testing, the test statistic (e.g. *F*, *t*, *r*) with confidence intervals, effect sizes, degrees of freedom and *P* value noted<br>*Give P values as exact values whenever suitable.*
☒	☐	For Bayesian analysis, information on the choice of priors and Markov chain Monte Carlo settings
☒	☐	For hierarchical and complex designs, identification of the appropriate level for tests and full reporting of outcomes
☐	☒	Estimates of effect sizes (e.g. Cohen's *d*, Pearson's *r*), indicating how they were calculated

Our web collection on statistics for biologists contains articles on many of the points above.

Software and code

Policy information about availability of computer code

Data collection	Confocal images were acquired on a Zeiss LSM 880 inverted confocal microscope (Carl Zeiss) using a Plan-Apochromat 63X objective (NA=1.4). Maximum intensity projections were generated with Zeiss ZEN Black software, and final composite images were created using ImageJ/Fiji (https://fiji.sc/). Live cell imaging was performed on a spinning-disk Nikon confocal microscope using a humidified environmental chamber kept at 37°C and 5% CO2, or with a Zeiss LSM 780 inverted confocal microscope (Carl Zeiss) fitted with a Plan-Apochromat 63X objective (NA=1.4).

Data analysis	Fiji/ImageJ NIH https://fiji.sc/ RRID:SCR_002285 v2.9.0<br>IMARIS Oxford Instruments https://imaris.oxinst.com/ RRID:SCR_007370 v8.2.0<br>GraphPad Prism GraphPad http://www.graphpad.com/ RRID:SCR_002798 v9.5.0<br>SnapGene Dotmatics http://www.snapgene.com/ RRID:SCR_015052 v6.2.1<br>BioRender BioRender https://www.biorender.com/ RRID:SCR_018361 v2023<br>AutoCAD Autodesk https://web.autocad.com/login v2023<br>STAR https://github.com/alexdobin/STAR v2.7.8a<br>featureCounts (subread) https://subread.sourceforge.net/ v2.0.1<br>R https://www.r-project.org/ v4.0.3<br>DESeq2 https://bioconductor.org/packages/release/bioc/html/DESeq2.html v1.30.1<br>ensembldb https://bioconductor.org/packages/release/bioc/html/ensembldb.html v2.14.0<br>clusterProfiler https://bioconductor.org/packages/release/bioc/html/clusterProfiler.html v3.18.1<br>GeneTonic https://bioconductor.org/packages/release/bioc/html/GeneTonic.html v1.5<br>Zen black https://www.micro-shop.zeiss.com/en/us/softwarefinder/software-categories/zenblack/ v14.0

For manuscripts utilizing custom algorithms or software that are central to the research but not yet described in published literature, software must be made available to editors and reviewers. We strongly encourage code deposition in a community repository (e.g. GitHub). See the Nature Portfolio guidelines for submitting code & software for further information.

Data

Policy information about availability of data

All manuscripts must include a data availability statement. This statement should provide the following information, where applicable:

- Accession codes, unique identifiers, or web links for publicly available datasets
- A description of any restrictions on data availability
- For clinical datasets or third party data, please ensure that the statement adheres to our policy

Reagents generated in this study are available upon request. All data are available in the main text or the supplementary materials. Further information and requests for resources and reagents should be directed to the lead contact, Juan S. Bonifacino (juan.bonifacino@nih.gov). Bulk RNA-Seq data have been deposited at GSE225479, and are publicly available as of the date of publication. Accession numbers are listed in the key resources table. Microscopy data reported in this paper will be shared by the lead contact upon request.

Research involving human participants, their data, or biological material

Policy information about studies with human participants or human data. See also policy information about sex, gender (identity/presentation), and sexual orientation and race, ethnicity and racism.

Reporting on sex and gender	N/A
Reporting on race, ethnicity, or other socially relevant groupings	N/A
Population characteristics	N/A
Recruitment	N/A
Ethics oversight	N/A

Note that full information on the approval of the study protocol must also be provided in the manuscript.

Field-specific reporting

Please select the one below that is the best fit for your research. If you are not sure, read the appropriate sections before making your selection.

☒ Life sciences ☐ Behavioural & social sciences ☐ Ecological, evolutionary & environmental sciences

For a reference copy of the document with all sections, see nature.com/documents/nr-reporting-summary-flat.pdf

Life sciences study design

All studies must disclose on these points even when the disclosure is negative.

Sample size	No statistical methods were used to predetermine sample sizes, but our sample sizes are similar to those reported in previous publications. (Maday et al. 2012; Cheng et al. 2015). Instead, multiple independent experiments were carried out using several sample replicates as detailed in the figure legends.
Data exclusions	Biological replicate #3 from WT Neurons and biological replicate #3 from WT Axons were outliers and therefore removed from for downstream differential expression analysis. This is specified in the text, in the Bioinformatic analyses section.

Replication	Multiple independent experiments were carried out using several sample replicates as detailed in the figure legends. All replication attempts were successful.
Randomization	No randomization was used when comparing i3Neurons wildtype and KO since treatment group of cells were generally derived from the same population of cells. For primary neuron analysis, animals from different litters were used for repeat experiments. Comparisons between KO and wildtype controls were made from neurons harvested within the same litter. In this study, animals were allocated into experimental groups based on genotype, so randomization was not applicable. For immunohistochemistry variation, animals or cells for the same experiment were always processed on the same day, using the same reagents, and were collected on the same slides for staining. Imaging parameters were held constant across the same experiment.
Blinding	A strategy for randomization, stratification or blind selection of samples was not carried out. Comparison between wildtype and KO neurons were not blinded for practical reasons as too few investigators were involved in the study.

Reporting for specific materials, systems and methods

We require information from authors about some types of materials, experimental systems and methods used in many studies. Here, indicate whether each material, system or method listed is relevant to your study. If you are not sure if a list item applies to your research, read the appropriate section before selecting a response.

Materials & experimental systems

n/a	Involved in the study
☐	☒ Antibodies
☐	☒ Eukaryotic cell lines
☒	☐ Palaeontology and archaeology
☐	☒ Animals and other organisms
☒	☐ Clinical data
☒	☐ Dual use research of concern
☒	☐ Plants

Methods

n/a	Involved in the study
☒	☐ ChIP-seq
☒	☐ Flow cytometry
☒	☐ MRI-based neuroimaging

Antibodies

Antibodies used

Primary antibodies:
BORCS5 (LOH12CR1), rabbit, used 1:500 for IB Proteintech Cat#17169-1-AP, RRID:AB_2137150
BORCS7 (C10orf32), rabbit, used 1:500 for IB Abnova Cat#PAB23142, RRID:AB_11122571
GAPDH (0411) HRP conjugated, used 1:1,000 for IB Santa Cruz Cat#sc-47724, RRID:AB_627678
Alpha-tubulin, mouse, used 1:5,000 for IB Santa Cruz Cat#sc-32293
RRID:AB_628412
LAMP1, mouse, used 1:500 for IF Developmental Studies Hybridoma Bank Cat#H4A3, RRID:AB_2296838
LAMP1, rat, used 1:500 for IB Developmental Studies Hybridoma Bank Cat#1D4B, RRID:AB_2134500
MAP2, chicken, used 1:1,000 for IB, 1:500 for IB Abcam Cat#ab5392, RRID:AB_2138153
MAP2 (H-300), rabbit, used 1:500 for IF Santa Cruz Cat#sc-20172, RRID:AB_2250101
Synaptophysin 1 (SYP1) (D-4), mouse, used 1:1,000 for IB Santa Cruz Cat#sc-17750, RRID:AB_628311
LAMTOR4 (C7orf59) (D4P6O), rabbit, used 1:500 for IF Cell Signaling Cat#13140, RRID:AB_2798129
Synaptic vesicle glycoprotein 2A (SV2), mouse, used 1:200 for IB, 1:200 for IF, 1:500 for IHC Developmental Studies Hybridoma Bank Cat#SV2, RRID:AB_2315387
TOMM20, rabbit, used 1:500 for IF, 1:200 for IB Proteintech Cat#11802-1-AP, RRID:AB_2207530
Tau-1, clone PC1C6, mouse, used 1:500 for IF Millipore Sigma Cat#MAB3420
Ankyrin-G, mouse, used 1:3 for IF NeuroMab Cat#73-146, RRID:AB_10697718
NDUFS1 (E4K3E), rabbit, used 1:100 for IB Cell Signaling Cat#70264
SDHA, mouse, used 1:100 for IB Abcam Cat#ab14715, RRID:AB_301433
CYCS (D18C7), rabbit, used 1:100 for IB Cell Signaling Cat#11940, RRID:AB_2637071
CYCS (6H2.B4), mouse, used 1:200 for IF BD Bioscience Cat#556432, RRID:AB_396416
COX IV (3E11), rabbit, used 1:100 for IB Cell Signaling Cat#4850, RRID:AB_2085424
ATP5A (15H4C4), mouse, used 1:100 for IB Abcam Cat#ab14748, RRID:AB_301447
MIC60/Mitofilin, rabbit, used 1:100 for IB Proteintech Cat#10179-1-AP, RRID:AB_2127193
MIC10/MINOS1, rabbit, used 1:100 for IB Novus Biologicals Cat#NBP1-91587, RRID:AB_11030043
LC3 (D11), rabbit, used 1:200 for IF Cell Signaling Cat#3868, RRID:AB_2137707
TGN46, sheep, used 1:1,000 for IF Bio-Rad Cat#AHP500G, RRID:AB_323104
Synapsin 1, rabbit, used 1:1,000 for IB Thermo Fisher Scientific Cat#PA1-4673, RRID:AB_561585
M6PR-CI (2G11), mouse, used 1:200 for IF Abcam Cat# ab2733, RRID:AB_2122792
EEA1(C45B10) rabbit, used 1:500 for IF Cell Signaling Cat#3288, RRID:AB_2096811
RPL24, rabbit, used 1:50 for IF Proteintech Cat# 17082-1-AP
RRID:AB_2181728
RPS27A, rabbit, used 1:50 for IF MyBioSource Cat#MBS7103451
Puromycin (12D10), mouse, 1:500 for IF Millipore Sigma Cat# MABE343
RRID:AB_2566826

Secondary antibodies:

Alexa Fluor 647-conjugated goat anti chicken IgY, used 1:1,000 for IF Thermo Fisher Scientific Cat#A-21449, RRID:AB_1500594
Alexa Fluor 555-conjugated donkey anti mouse IgG, used 1:1,000 for IF Thermo Fisher Scientific Cat#A-31570, RRID:AB_2536180
Alexa Fluor 488-conjugated donkey anti rabbit IgG, used 1:1,000 for IF Thermo Fisher Scientific Cat#A-21206, RRID:AB_2535792
Alexa Fluor 647-conjugated goat anti sheep IgG, used 1:1,000 for IF Thermo Fisher Scientific Cat#A-21448, RRID:AB_2535865
HRP-conjugated goat anti-rabbit IgG (H+L), used 1:5,000 for IF Jackson ImmunoResearch Cat#111-035-144, RRID:AB_2307391
HRP-conjugated goat anti-chicken IgY (H+L), used 1:5,000 for IF Jackson ImmunoResearch Cat#103-035-155, RRID:AB_2337381
HRP-conjugated donkey anti-mouse IgG (H+L), used 1:5,000 for IF Jackson ImmunoResearch Cat#715-035-150, RRID:AB_2340770

Validation

All antibodies are from commercially available sources and have been validated by the manufacturer with supporting publications found on manufacturer websites.
For rabbit BORCS5 (LOH12CR1): https://www.ptgcn.com/products/LOH12CR1-Antibody-17169-1-AP.htm
For rabbit BORCS7 (C10orf32): https://www.abnova.com/en-global/product/detail/pab23142
For mouse GAPDH (0411): https://www.scbt.com/p/gapdh-antibody-0411
For mouse Alpha-tubulin: https://www.scbt.com/p/alpha-tubulin-antibody-dm1a
For mouse LAMP1: https://dshb.biology.uiowa.edu/H4A3
For Rat LAMP1: https://dshb.biology.uiowa.edu/1D4B
For chicken MAP2: https://www.abcam.com/products/primary-antibodies/map2-antibody-ab5392.html
For rabbit MAP2(H-300): https://www.scbt.com/p/map-2-antibody-h-300
For mouse Synaptophysin 1 (SYP1) (D-4): https://www.scbt.com/p/syp-antibody-d-4
For rabbit LAMTOR4 (C7orf59) (D4P6O): https://www.cellsignal.com/products/primary-antibodies/lamtor4-c7orf59-d4p6o-rabbit-mab/13140
For mouse Synaptic vesicle glycoprotein 2A (SV2): https://dshb.biology.uiowa.edu/SV2
For rabbit TOMM20: https://www.ptglab.com/products/TOM20-Antibody-11802-1-AP.htm
For mouse Tau-1, clone PC1C6: https://www.sigmaaldrich.com/US/en/product/mm/mab3420
For mouse Ankyrin-G: https://www.antibodiesinc.com/products/anti-ankyrin-g-staining-antibody-n106-36-75-146
For rabbit NDUFS1 (E4K3E): https://www.cellsignal.com/products/primary-antibodies/ndufs1-e4k3e-rabbit-mab/70264
For mouse SDHA: https://www.abcam.com/products/primary-antibodies/sdha-antibody-2e3gc12fb2ae2-ab14715.html
For rabbit CYCS (D18C7): https://www.cellsignal.com/products/primary-antibodies/cytochrome-c-d18c7-rabbit-mab/11940
For mouse CYCS (6H2.B4): https://www.bdbiosciences.com/en-us/products/reagents/microscopy-imaging-reagents/immunofluorescence-reagents/purified-mouse-anti-cytochrome-c.556432
For rabbit COX IV (3E11): https://www.cellsignal.com/products/primary-antibodies/cox-iv-3e11-rabbit-mab/4850
For mouse ATP5A (15H4C4): https://www.abcam.com/products/primary-antibodies/atp5a-antibody-15h4c4-mitochondrial-marker-ab14748.html
For rabbit MIC60/Mitofilin: https://www.ptglab.com/products/IMMT-Antibody-10179-1-AP.htm
For rabbit MIC10/MINOS1: https://www.novusbio.com/products/minos1-antibody_nbp1-91587
For rabbit LC3 (D11): https://www.cellsignal.com/products/primary-antibodies/lc3b-d11-xp-rabbit-mab/3868
For sheep TGN46: https://www.bio-rad-antibodies.com/polyclonal/human-tgn46-antibody-ahp500.html?f=purified
For rabbit Synapsin 1: https://www.thermofisher.com/antibody/product/Synapsin-1-Antibody-Polyclonal/PA1-4673
For mouse M6PR-CI (2G11): https://www.abcam.com/products/primary-antibodies/m6pr-cation-independent-antibody-2g11-ab2733.html
For rabbit EEA1(C45B10): https://www.cellsignal.com/products/primary-antibodies/eea1-c45b10-rabbit-mab/3288
For rabbit RPL24: https://www.ptglab.com/products/RPL24-Antibody-17082-1-AP.htm
For rabbit RPS27A: https://www.mybiosource.com/polyclonal-human-antibody/rps27a/7103451
For mouse Puromycin (12D10): https://www.sigmaaldrich.com/US/en/product/mm/mabe343
For Alexa Fluor 647-conjugated goat anti chicken IgY: https://www.thermofisher.com/antibody/product/Goat-anti-Chicken-IgY-H-L-Secondary-Antibody-Polyclonal/A-21449
For Alexa Fluor 555-conjugated donkey anti mouse IgG: https://www.thermofisher.com/antibody/product/Donkey-anti-Mouse-IgG-H-L-Highly-Cross-Adsorbed-Secondary-Antibody-Polyclonal/A-31570
For Alexa Fluor 488-conjugated donkey anti rabbit IgG: https://www.thermofisher.com/antibody/product/Donkey-anti-Rabbit-IgG-H-L-Highly-Cross-Adsorbed-Secondary-Antibody-Polyclonal/A-21206
For Alexa Fluor 647-conjugated goat anti sheep IgG: https://www.thermofisher.com/antibody/product/Donkey-anti-Sheep-IgG-H-L-Cross-Adsorbed-Secondary-Antibody-Polyclonal/A-21448
For HRP-conjugated goat anti-rabbit IgG (H+L): https://www.jacksonimmuno.com/catalog/products/111-035-144
For HRP-conjugated goat anti-chicken IgY (H+L): https://www.jacksonimmuno.com/catalog/products/103-035-155
For HRP-conjugated donkey anti-mouse IgG (H+L): https://www.jacksonimmuno.com/catalog/products/715-035-150

Eukaryotic cell lines

Policy information about cell lines and Sex and Gender in Research

Cell line source(s)

Induced pluripotent stem cells (iPSCs) expressing the neuronal transcriptional activator neurogenin 2 (NGN2) under the control of a doxycycline-inducible promoter were obtained from Michael E. Ward lab, and generated as previously described (Wang C et al. Stem Cell Reports. 2017; 9: 1221-1233; Fernandopulle MS et al. Curr Protoc Cell Biol. 2018; 79: e51). HEK-293T cells were bought from ATCC Cat#CRL-11268.

Authentication

Specific KO mutations were confirmed by PCR amplification followed by Sanger sequencing.

Mycoplasma contamination

All cell lines tested negative for mycoplasma contamination.

Commonly misidentified lines
(See ICLAC register)

No commonly misidentified cell lines were used.

Animals and other research organisms

Policy information about studies involving animals; ARRIVE guidelines recommended for reporting animal research, and Sex and Gender in Research

Laboratory animals	All mice used in this study have C57BL/6J background. Mice used in the study were naïve and had normal health status. For timed pregnancies, we housed single male and female mice of 6 weeks of age in separate cages under a 12-h light-dark cycle. Housing conditions were maintained at ~23°C with 40-60% humidity. To time the deliveries, the female's weight was recorded, and the female was transferred overnight into the cage housing the male for no longer than 12 h. The following day, the two animals were separated, and the female was checked for the presence of plaque. If the plaque was absent, the female was transferred again to the male cage the following night. We monitored the weight of the female to determine increases over time. When the pregnancy was successful, we harvested embryos after 17 days for culture of primary cortical neurons. Neurons of mice with identical genotype and from the same litter were pooled and analyzed.
Wild animals	This study did not involve wild animals.
Reporting on sex	Embryonic harvesting of primary neurons did not take in consideration the sex of the animals because it cannot be easily determined at this developmental stage.
Field-collected samples	This study did not include samples collected from the field.
Ethics oversight	All mouse procedures were conducted following the NIH Guide for the Care and Use of Laboratory Animals, under protocol #21-021 approved by the NICHD Animal Care and Use Committee.

Note that full information on the approval of the study protocol must also be provided in the manuscript.
