# Supplementary figures and images for: Messenger RNA transport on lysosomal vesicles maintains axonal mitochondrial homeostasis and prevents axonal degeneration
Source: Nat Neurosci. 2024 Apr 10;27(6):1087–102. doi: 10.1038/s41593-024-01619-1 (PMC11156585; doi:10.1038/s41593-024-01619-1)

Fig. 1 c

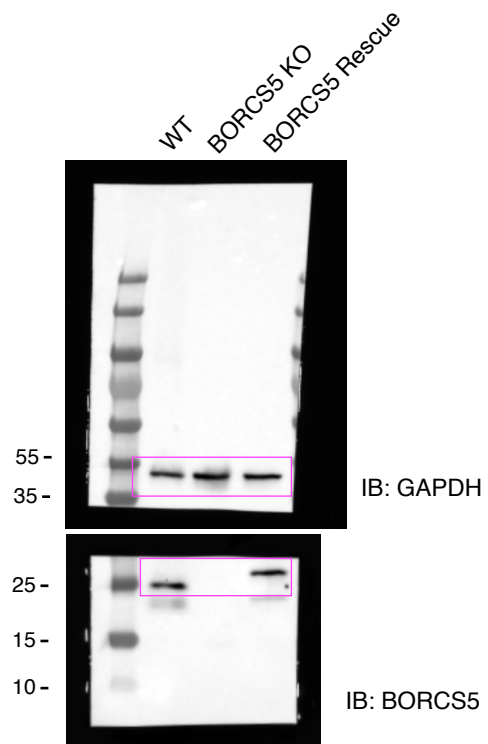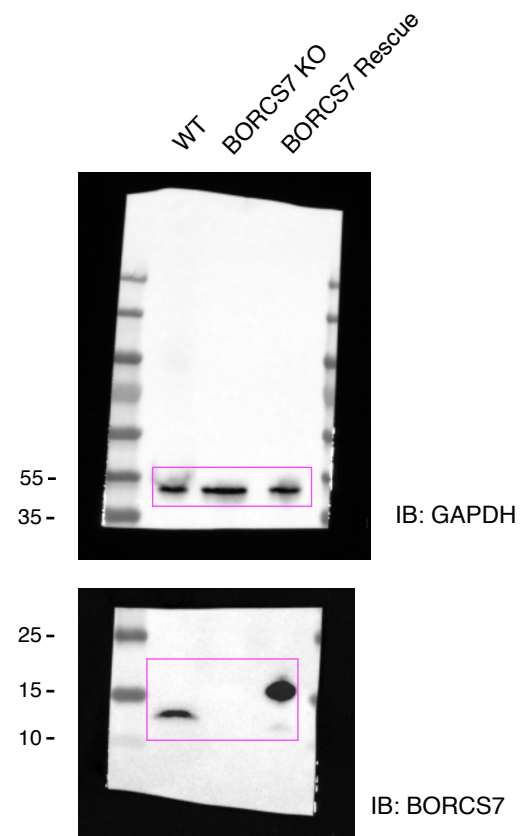

Supplement: Supplementary file 9 — Unprocessed western blots. [file 41593_2024_1619_MOESM9_ESM.pdf]

Fig. 2 d

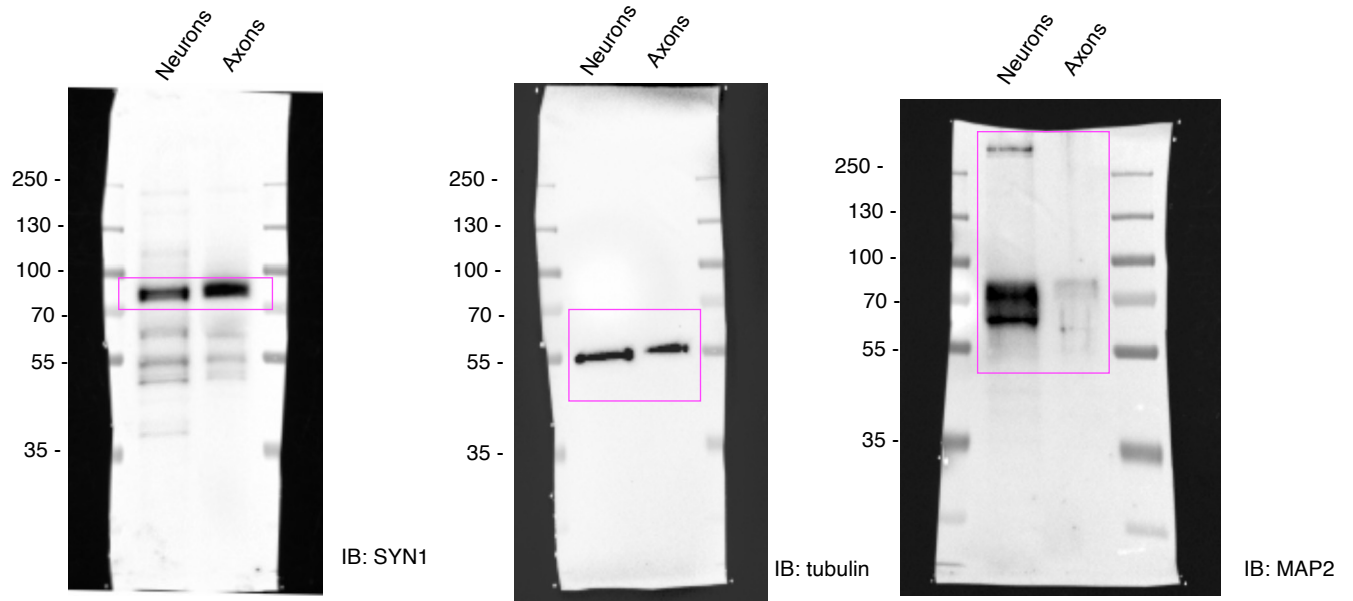

Supplement: Supplementary file 10 — Unprocessed western blots. [file 41593_2024_1619_MOESM10_ESM.pdf]

Fig. 6 a

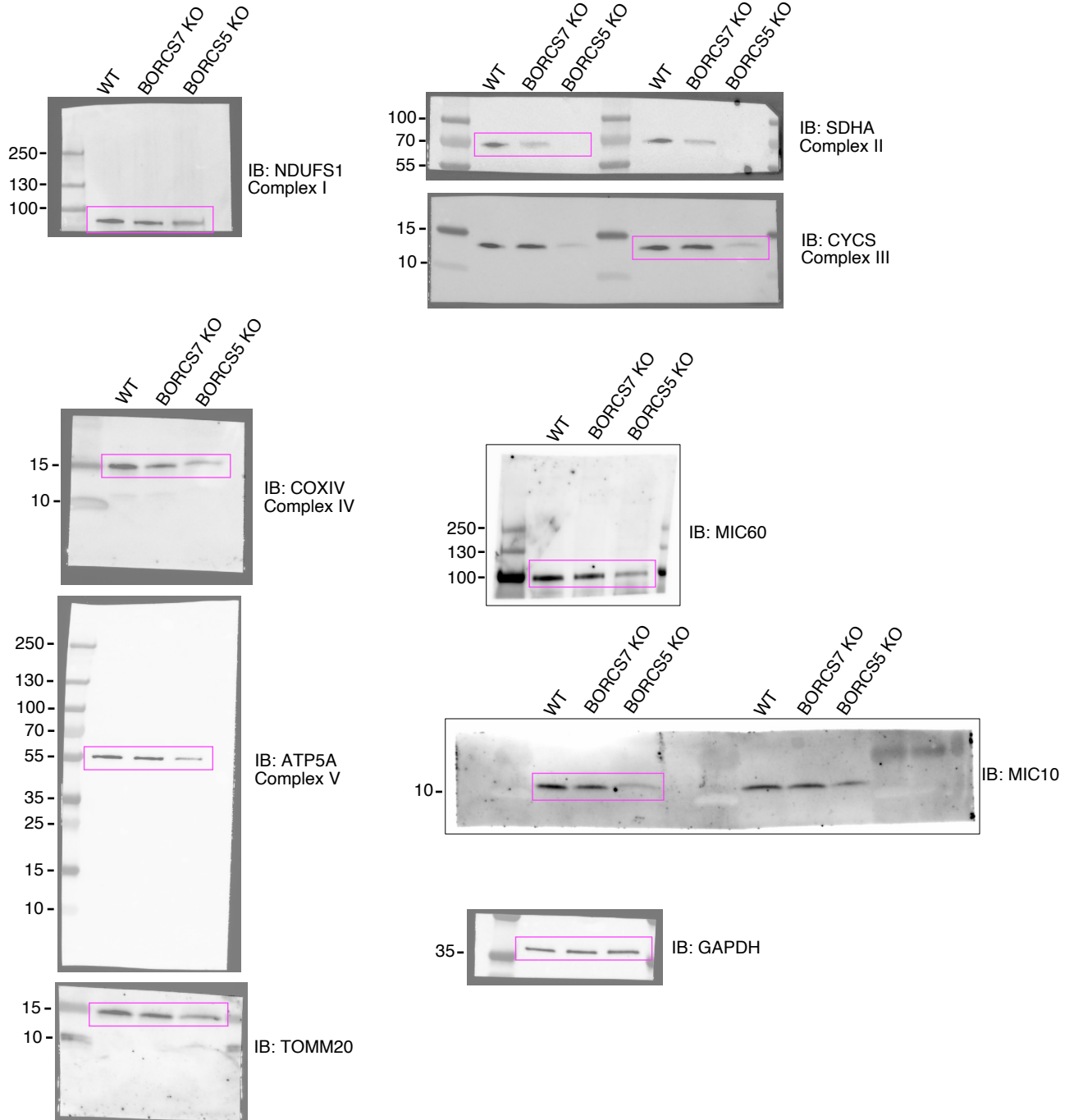

Supplement: Supplementary file 11 — Unprocessed western blots. [file 41593_2024_1619_MOESM11_ESM.pdf]
